# Ndfip2 in TrkA-expressing sensory neurons regulates noxious mechanosensation through control of TrkA signaling and protein levels

**DOI:** 10.1038/s41419-026-08670-9

**Published:** 2026-03-31

**Authors:** Daniel Cañada-García, Ana Hernández-García, Cristina Vicente-García, Jorge Valero, Maurilyn Ayon-Olivas, Sharad Kumar, Michael Sendtner, Juan Carlos Arévalo

**Affiliations:** 1 Department of Cell Biology and Pathology, Instituto de Neurociencias de Castilla y León (INCyL), Universidad de Salamanca, Salamanca, Spain; 2 Institute of Biomedical Research of Salamanca (IBSAL), Salamanca, Spain; 3 Institute of Clinical Neurobiology, University Hospital Wuerzburg, Wuerzburg, Germany; 4 Centre for Cancer Biology, College of Heath, Adelaide University, Adelaide, SA Australia

**Keywords:** Somatic system, Neurotrophic factors

## Abstract

Nociception, the neural process underlying pain detection, is modulated by the NGF/TrkA signaling axis. Although anti-NGF antibodies can alleviate chronic pain, their clinical application is limited by adverse effects, underscoring the need to identify downstream regulators of this pathway. One such mechanism involves TrkA ubiquitination mediated by Nedd4 E3 ubiquitin ligases, whose activity is modulated by Nedd4 family interacting protein 2 (Ndfip2). Notably, Ndfip2 expression is regulated by TrkA signaling under pain conditions. Here, we characterize the physiological and molecular roles of Ndfip2 in sensory neurons. We demonstrate that Ndfip2 localizes to the endoplasmic reticulum and Golgi apparatus and interacts with TrkA in sensory neurons. Conditional deletion of Ndfip2 in TrkA-expressing cells selectively alters mechanical nociception. Mechanistically, loss of Ndfip2 decreases total TrkA protein levels, downstream activation, and cell-surface exposition, particularly in male-derived dorsal root ganglia neurons. Conversely, Ndfip2 expression reduces mature glycosylated TrkA and promotes the accumulation of non-glycosylated forms, consistent with impaired receptor maturation. Together, these findings identify Ndfip2 as a post-translational regulator of TrkA in TrkA-lineage sensory neurons and establish its in vivo role in mechanical nociception.

## Introduction

Pain functions as an essential alarm and protective mechanism in response to noxious stimuli, which are detected and encoded through a process known as nociception. Nociception is initiated by specialized sensory neurons, nociceptors, located in the dorsal root ganglia (DRG) or trigeminal ganglia (TG). These nociceptors detect and respond to a variety of harmful stimuli, with most being polymodal in nature [1]. The development and functional specification of nociceptors are primarily governed by neurotrophin signaling, particularly through the interaction of nerve growth factor (NGF) with its high-affinity receptor TrkA [2]. Activation of this axis triggers downstream signaling cascades, such as PLC-γ or ERK1/2, which in turn regulate the expression of genes involved in nociceptive processing [3].

The NGF/TrkA axis plays a critical role in pain modulation, as demonstrated by multiple lines of evidence: (1) NGF expression is elevated in patients with knee osteoarthritis [4]; (2) mutations in the human *NTRK1* gene, which encodes TrkA, cause congenital insensitivity to pain with anhidrosis (CIPA), a rare sensory neuropathy [5]; and (3) clinical trials targeting NGF with monoclonal antibodies such as Tanezumab and Fasinumab have shown significant analgesic effects in osteoarthritis patients [6, 7]. However, adverse effects observed in these trials have limited the clinical applicability of anti-NGF therapies. Consequently, there is an urgent need to identify novel molecules and signaling mechanisms downstream of the NGF/TrkA pathway that can provide effective pain relief while minimizing side effects.

The function of the NGF/TrkA axis is tightly regulated by post-translational mechanisms, including ubiquitination of the TrkA receptor. Among several E3 ubiquitin ligases, Nedd4-2 has been identified as a key regulator of TrkA ubiquitination [8]. Upon NGF binding, TrkA signaling is attenuated through Nedd4-2-mediated ubiquitination, which targets the receptor for lysosomal degradation [8]. When TrkA ubiquitination is disrupted, as in genetic models, sustained receptor activation results in enhanced nociceptive responses [9]. Using a TrkA mutant mouse model with impaired ubiquitination and increased signaling [10], we previously conducted an unbiased transcriptomic screening to identify downstream effectors of NGF/TrkA signaling under pain conditions. Among the candidates, Nedd4 family interacting protein 2 (Ndfip2) emerged as a promising regulator of this pathway [3].

Ndfip proteins (Ndfip1 and Ndfip2) function as adaptor molecules that modulate the activity of Nedd4 family E3 ubiquitin ligases through their localization, catalytic activity and E3-substrate interaction. At the same time, both can be ubiquitinated as potential substrates for Nedd4 ligases [11, 12]. They are localized in the Golgi and Golgi-derived vesicles and endosomes, and Ndfip2 has also been described in endoplasmic reticulum (ER) and multivesicular bodies in cell lines [12–15]. Ndfip2 activates the catalytic domain of Nedd4 ligases to facilitate ubiquitin transfer [16–20], inhibits Nedd4-mediated ubiquitination of certain substrates [13, 21], and regulates Nedd4 protein levels [22]. Additionally, Ndfips enable Nedd4 ligases to ubiquitinate substrates lacking canonical PY motifs, thereby expanding their substrate repertoire [15, 16, 20, 23–27]. Of particular interest is the Nedd4-2–mediated ubiquitination of TrkB through Ndfip1, which promotes receptor trafficking to late endosomes and subsequent degradation via the lysosomal pathway [23]. Despite the importance of ubiquitination in neurotrophic signaling regulation and Ndfip proteins regulatory role in Nedd4 activity, it remains unclear whether Ndfip proteins contribute to the regulation of NGF/TrkA signaling.

In this study, we investigated the role of Ndfip2 in the regulation of the NGF/TrkA signaling axis. We found that Ndfip2 localizes to the ER and Golgi apparatus (GA) in sensory neurons and interacts with TrkA, but not with Nedd4-2. Conditional deletion of Ndfip2 led to a reduction in total TrkA protein levels, an effect that was more pronounced in male-derived DRG neurons. In vivo, mice with a conditional deletion of Ndfip2 in TrkA-expressing cells exhibited decreased sensitivity to noxious mechanical stimulation. Mechanistically, we show that Ndfip2 post-translationally modulates TrkA levels, activation, and glycosylation. These findings indicate that Ndfip2 expressed in TrkA-expressing DRG neurons regulates mechanical pain by modulating the levels and function of TrkA.

## Results

### Ndfip2 is localized to the endoplasmic reticulum and Golgi apparatus in DRG neurons and interacts with TrkA but not with Nedd4-2

**Fig. 1:**
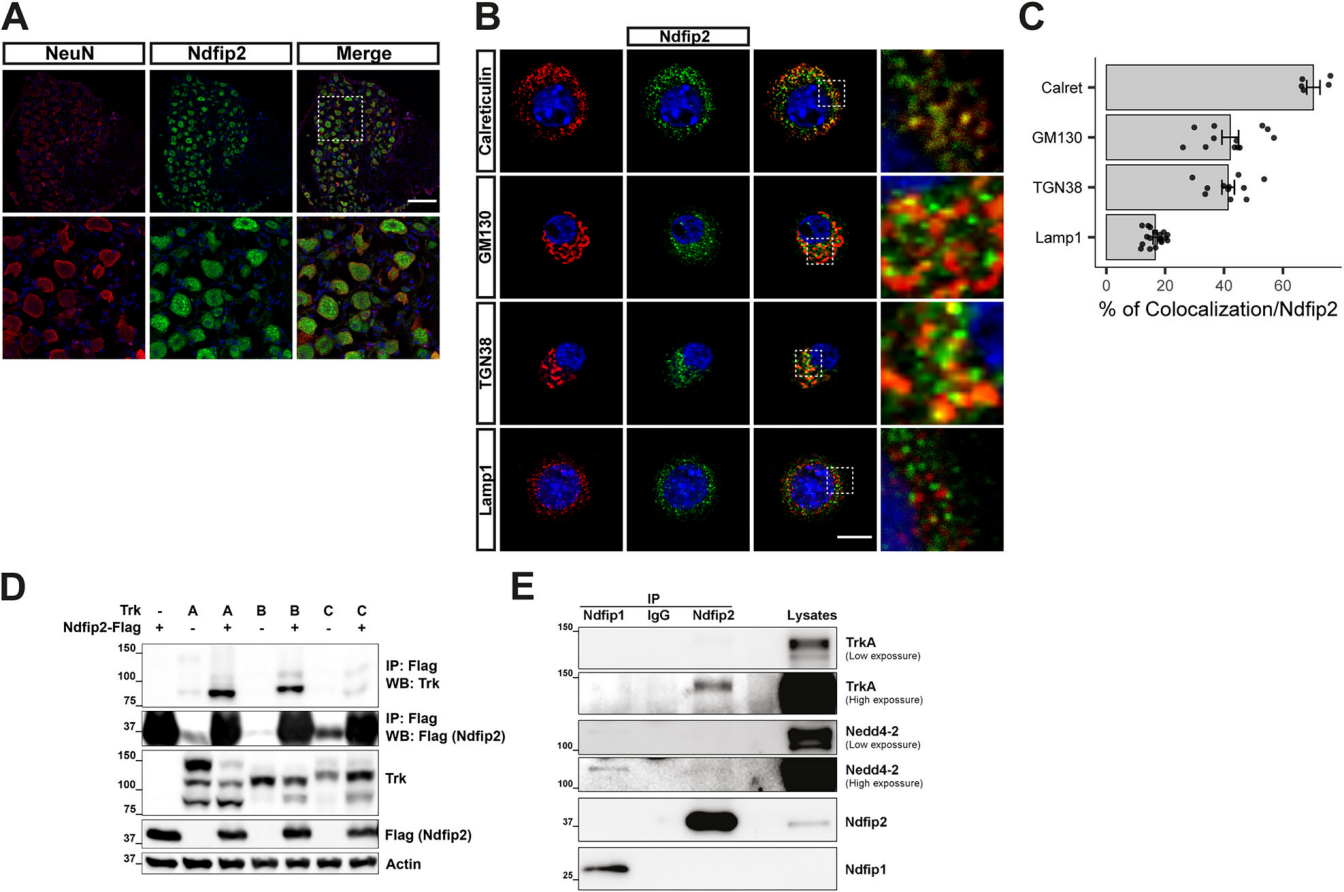
Ndfip2 is localized at the endoplasmic reticulum and Golgi apparatus in DRG neurons and interacts with TrkA. **A** Adult mouse L3 DRG sections were stained with NeuN and Ndfip2 antibodies. Images were captured using a confocal microscope. Representative pictures are shown (*n* = 3 mice, 3-4 images per mouse). Scale bar, 100 µm. **B** Co-localization of Ndfip2 (green) with calreticulin, GM130, TGN38, and Lamp1 (red) in cultured DRG neurons. Representative images are shown Scale bar, 10 µm. **C** Quantification of the staining (*n* = 5, 12, 11, 19 cells/staining). Note the preferential location of Ndfip2 in ER. **D** Ndfip2 interacts with TrkA, TrkB and TrkC receptors in transfected HEK293FT cells. A representative Western blot is shown (*n* = 3). **E** Ndfip2 interacts with TrkA and Ndfip1 with Nedd4-2 in cultured DRG neurons. A representative experiment is shown (*n* = 7).

**Fig. 2:**
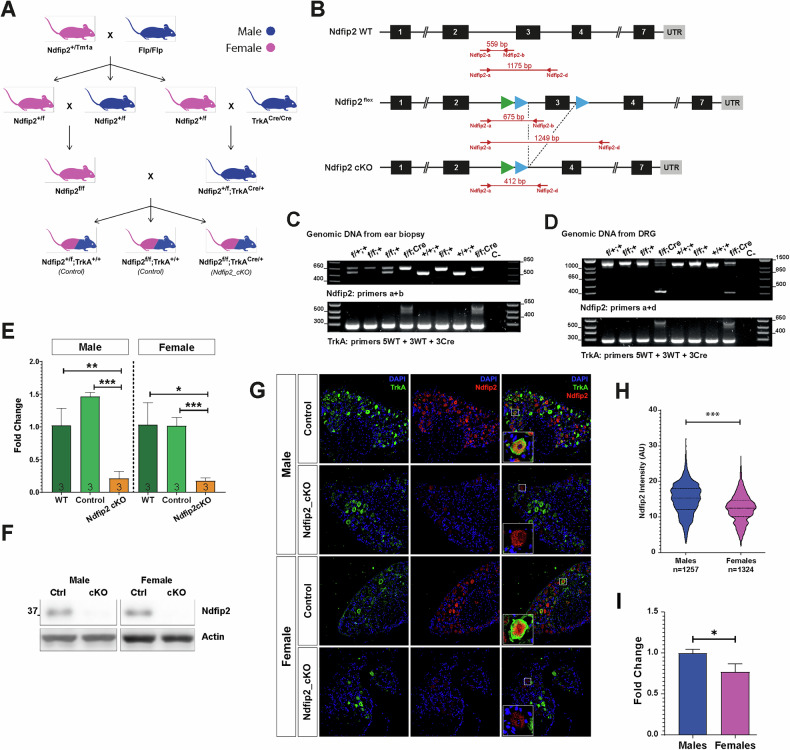
Generation and validation of *Ndfip2* KO in TrkA-expressing DRG neurons. **A** Picture depicting the breeding strategy to generate *Ndfip2* conditional KO (cKO) in TrkA-expressing cells. **B** Scheme showing the genomic sequences and PCR amplicons obtained for each allele of wild type Ndfip2, *Ndfip2*^*f/f*^ and *Ndfip2* cKO as result of Cre recombination. **C** In the upper panel, representative PCR genotyping using genomic DNA from ears for *Ndfip2*^*+/+*^ (lanes 5 and 7), *Ndfip2*^*f/f*^ (lanes 2, 4, 6, and 8) and *Ndfip2*^*f/+*^ (lanes 1 and 3). In the lower panel, representative PCR genotyping results using DNA from DRGs for *TrkA*^*+/+*^ (lanes 1–3 and 5–7) and *TrkA*^*Cre/+*^ (lanes 4 and 8). **D** Representative PCR results from genomic DNA of DRGs to validate recombination in *Ndfip2*^*f/f*^;*TrkA*^*Cre/+*^ mice (lanes 4 and 8 show an amplicon of 412 bp). For **C**, **D** primers used for each PCR are indicated. **E** RT-qPCR validation of *Ndfip2* mRNA reduction in *Ndfip2*^*f/f*^*;* *TrkA*^*Cre/+*^ mice, also confirming that the *Ndfip2*^*f/f*^ (control) behaves similarly to the WT (*n* = 3). **F** Ndfip2 depletion in cultured TrkA-expressing DRG neurons from E13.5 *Ndfip2* cKO embryos. Representative Western blots from male and female DRG cultures are shown. Actin was used as loading control. **G** Specific Ndfip2 depletion in TrkA-expressing neurons from adult *Ndfip2* cKO DRGs. Representative immunofluorescence of male and female L3 DRGs shows that in the *Ndfip2* cKO samples, TrkA-expressing neurons (green) lack Ndfip2 staining (red), which is observed in control animals and in TrkA-negative neurons. Note that in *Ndfip2* cKO samples, Ndfip2 positive cells do not express TrkA. Scale bar, 100 µm. **H** Quantification of Ndfip2 intensity staining in male and female control mice, showing the sexual dimorphism (*n* = 1257 and 1324 for males and females, respectively). **I** RT-qPCR of *Ndfip2* mRNA in control male and female mice (*n* = 3).

Ndfip2 is expressed in the mouse spinal cord and DRG during development at E11.5 [16], but its expression in the adult DRG is unknown. Using the neuronal marker NeuN, we found that Ndfip2 is expressed in adult DRGs and restricted to neurons (Fig. 1A). Within the neuronal population, Ndfip2 was detected in TrkA-positive neurons (Fig. 2G), which correspond to nociceptors, and displays a punctate staining pattern (Fig.1A). Colocalization analysis in cultured TrkA-expressing DRG neurons revealed that Ndfip2 was mainly located at the ER and the GA, and residual localization at lysosomes (Fig. 1B, C). Given that Ndfip2 acts as an adaptor protein for Nedd4-2 [12], which is known to interact with and regulate TrkA [8, 9], we investigated whether Ndfip2 is associated with TrkA. In transfected HEK293FT cells, we found that Ndfip2 interacted not only with TrkA but also with TrkB and TrkC receptors (Fig. 1D). In cultured rat DRG neurons, Ndfip2 was associated with TrkA but, surprisingly, not to Nedd4-2 (Fig. 1E). However, Ndfip1 interacted with Nedd4-2, but not with TrkA (Fig. 1E). Together, these findings indicate that Ndfip2 localizes to the ER and GA and interacts with TrkA in DRG neurons.

### Generation of an *Ndfip2* conditional knockout mouse in TrkA-expressing cells

To investigate the physiological role of Ndfip2 in TrkA-expressing cells, we generated a conditional knockout (cKO) mouse by crossing *Ndfip2*^*f/f*^ mice with *TrkA*^*Cre/+*^ mice (Fig. 2A). Genotyping by PCR confirmed the presence of both the Cre recombinase and floxed Ndfip2 alleles (Fig. 2B, C), with evidence of partial, tissue-specific recombination in the DRGs (Fig. 2D). Furthermore, RT-qPCR analysis from DRGs showed a significant reduction in *Ndfip2* mRNA levels in cKO mice compared to both wild-type (WT) and control (*Ndfip2*^*f/f*^) animals, with no difference between the latter (Fig. 2E). To validate loss of Ndfip2 protein, we used cultured TrkA-expressing DRG neurons, which showed complete loss of Ndfip2 protein in cKO samples (Fig. 2F). Consistently, immunofluorescence analysis on adult DRG sections confirmed the absence of Ndfip2 staining in TrkA-expressing neurons from both male and female cKO mice, while Ndfip2 positive neurons are TrkA negative (Fig. 2G). Interestingly, immunofluorescence quantification showed that control male mice displayed higher Ndfip2 levels than females, revealing a sex-dependent difference in basal Ndfip2 expression (Fig. 2H). RT-qPCR analysis showed a lower Ndfip2 mRNA expression in females (Fig. 2I), suggesting a transcriptional effect. Altogether, these results confirm the successful generation of an *Ndfip2* cKO model in TrkA-expressing DRG neurons, providing a valuable tool to explore the role of Ndfip2 in sensory neuron function.

### Ndfip2 deletion in sensory neurons impairs mechanical nociception

Functional specification of nociceptors during development is controlled by NGF/TrkA signaling [28]. To investigate the impact of selective Ndfip2 deletion on nociception, we subjected *Ndfip2* cKO mice to a series of behavioral and sensory assays. Considering the reported sexual dimorphism in nociceptive behaviors [29], in the neuronal populations in the DRGs [30], and in Ndfip2 expression described above (Fig. 2H, I), males and females were analyzed separately. No significant differences were observed between control and *Ndfip2* cKO mice in general locomotion and anxiety-related behavior, as assessed by total distance traveled (Fig. S3A), mean speed (Fig. S3B), and time spent in the center of the arena (Fig. S3C). This indicates that Ndfip2 deletion in TrkA-expressing cells does not influence general locomotion or anxiety-like behavior, ensuring that subsequent analyses are not influenced by these factors.

**Fig. 3:**
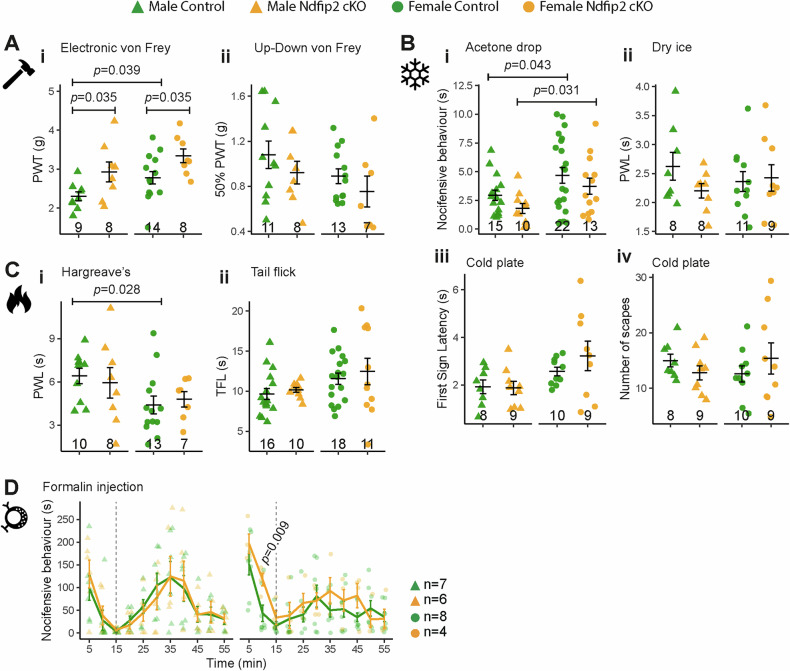
Ndfip2 deletion alters mechanical pain and nocifensive response to phase I in formalin test. **A** i *Ndfip2* cKO male and female mice show hyposensitivity to mechanical pain; ii *Ndfip2* cKO mice display similar threshold to mechanical nociception as control littermates in the up-down von Frey test. **B** i *Ndfip2* cKO mice behave like controls in the acetone evaporation test; ii *Ndfip2* cKO mice respond like control mice to noxious cold in the dry ice test; iii, iv control and *Ndfip2* cKO mice respond similarly in the latency to display the first cold response and in the number of trials to escape. **C** Similar response in control and *Ndfip2* cKO mice to the Hargreaves’ test (i) and to the tail flick test (ii). **D** i Nocifensive response of *Ndfip2* cKO females (right panel) is enhanced at 10 min compared to control mice, whereas *Ndfip2* cKO males (left panel) behave similarly to control male mice. The graphs show the mean ± SEM of the number of animals tested for each group (bottom each graph and right for (**D**) and statistical significance was evaluated using a t-test, with Welch correction where appropriate (See also Supplementary Data 1).

Then we evaluate the role of Ndfip2 in mechanical nociception. In response to electronic von Frey, both male and female *Ndfip2* cKO mice exhibited increased paw withdrawal thresholds, indicating reduced mechanical pain sensitivity (Fig. 3Ai, Supplementary Data 1). However, no significant differences were found between groups using the manual von Frey up-and-down method (Fig. 3Aii, Supplementary Data 1). Therefore, Ndfip2 contributes to mechanical pain processing.

To assess cold perception, we employed several behavioral assays. In the acetone evaporation test for cooling, *Ndfip2* cKO mice did not exhibit statistically significant differences in nocifensive responses compared to controls, although a moderate hyposensitivity was observed in males (*ĝ*_Hedges_ = 0.70, *p* = 0.08) (Fig. 3Bi; Supplementary Data 1). Conversely, a moderate, but not statistically significant, hypersensitivity was detected in *Ndfip2* cKO male mice in the dry ice test (*ĝ*_Hedges_ = 0.72, *p* = 0.14) (Fig. 3Bii; Supplementary Data 1). No relevant differences were observed in the cold plate test (Fig. 3Biii, iv; Supplementary Data 1). Therefore, Ndfip2 influences moderately cold behavior in male mice.

Heat sensitivity was assessed using Hargreaves’ and tail flick tests. *Ndfip2* cKO and control mice showed similar latencies in both assays (Fig. 3Ci, ii, Supp. Data 1), suggesting that thermal nociception remains unaltered upon Ndfip2 deletion.

To explore inflammatory pain responses, we conducted the formalin test. Female *Ndfip2* cKO mice exhibited increased sensitivity during phase I of the formalin response, showing a significant increase in nocifensive behavior 10 minutes after injection (Fig. 3D and Supplementary Data 1). No significant differences were observed during the inflammatory phase II in either sex (Fig. 3D and Supplementary Data 1). Thus, Ndfip2 deletion enhances acute inflammatory pain in females.

When comparisons were performed between sexes, we observed significant differences in control mice in the mechanical pain (Fig. 3A) and thermal nociception (Fig. 3C) and in control and *Ndfip2* KO mice in the acetone cold test (Fig. 3B). Collectively, our findings demonstrate that Ndfip2 contributes mainly to mechanical pain processing, and its deletion has sex-dependent effects on non-noxious cold responses and inflammatory pain.

### Ndfip2 deletion alters TrkA levels in male adult DRGs

**Fig. 4:**
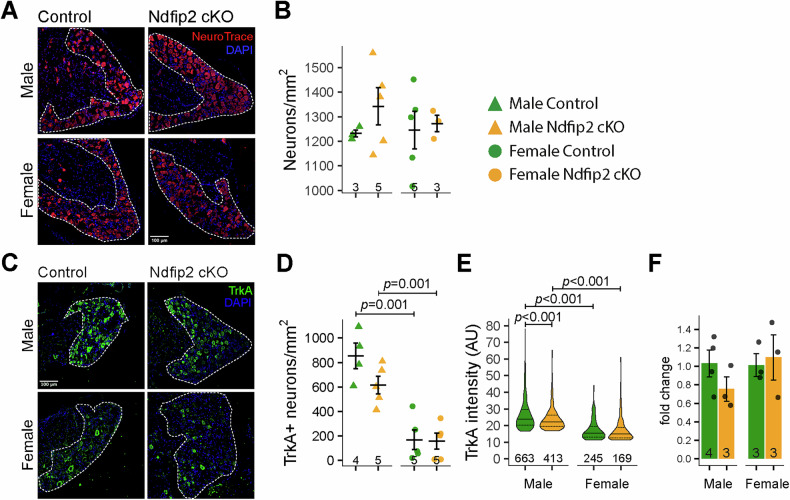
Deletion of Ndfip2 reduces TrkA levels in male DRG neurons. **A** Representative immunofluorescence staining of L3 DRG neurons from control and *Ndfip2* cKO male and female mice using NeuroTrace. Scale bar, 100 µm. **B** Quantification of the number of neurons per square millimeter. The area quantified is shown within the dashed line in (**A**). Each point is the average of 4-8 different images (*n* = 3–5). **C** L3 DRG sections obtained from male and female control and Ndfip2 cKO were stained with TrkA antibodies. Scale bar, 100 µm. **D** Quantification of the number of TrkA-positive neurons. Phosphorylated PLCγ calculated as panel C (left panel). PLCγ activation dynamics similar as shown in panel C (right panel). **E** Quantification of TrkA intensity staining in male and female control and *Ndfip2* cKO DRGs (*n* = 3 animals, with 663, 413, 245, and 169 positive neurons for control and *Ndfip2* cKO male and female DRGs, WMW test). Note the significant decrease in TrkA intensity in male *Ndfip2* cKO and the sexual dimorphism for both genotypes. **F** RT-qPCR showing similar TrkA mRNA levels in control and *Ndfip2* cKO mice (*n* = 3, 4).

To determine whether this behavioral phenotype reflects changes in the DRGs, we analyzed adult DRG sections from control and *Ndfip2* cKO mice. Total neuronal density did not differ significantly between genotypes (Fig. 4A, B).

To specifically examine the neuronal populations underlying nociceptive changes, we quantified TrkA-immunolabeled sections. Comparison between genotypes showed a large, though not statistically significant, reduction in male *Ndfip2* cKO density (*ĝ*_Hedges_ = 1.11, *p* = 0.09) (Fig. 4C, D) and a significant decrease in TrkA intensity in mutant male mice (*rrb* = 0.16, *p* = 1.83e-05; Fig. 4C, E). No genotype-dependent differences were detected in females for either neuronal density or staining intensity. A significant reduction in the number of TrkA-positive neurons and TrkA intensity in control and *Ndfip2* cKO females compared with males was observed (Fig. 4D, E). RT-qPCR analysis showed no differences in *TrkA* mRNA levels (Fig. 4F), pointing to post-transcriptional rather than transcriptional mechanisms the effects observed at the TrkA protein level. Together, these results indicate that Ndfip2 selectively regulates TrkA protein levels in male DRG neurons.

### Ndfip2 deletion attenuates NGF signaling mainly in male DRG neurons

**Fig. 5:**
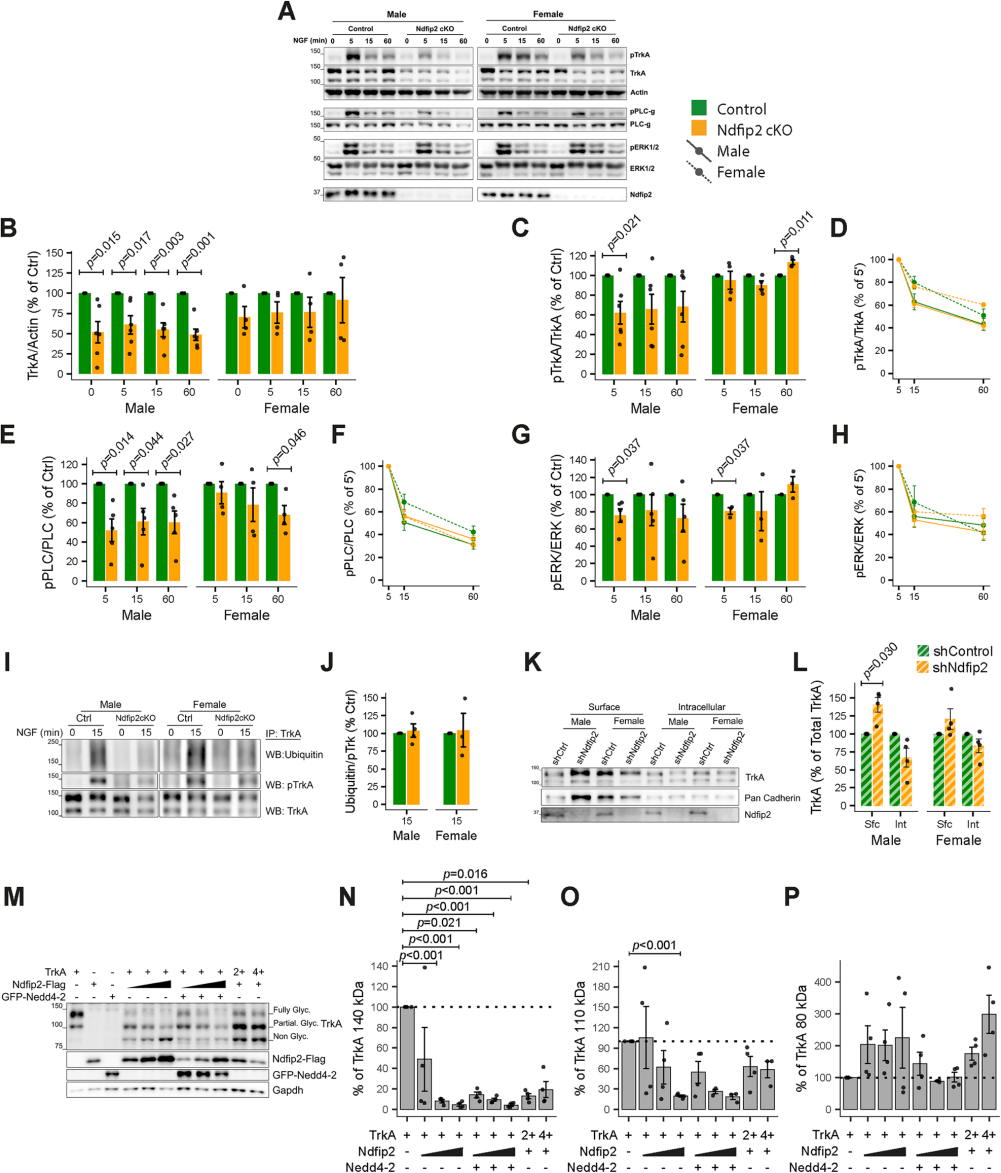
Deletion of Ndfip2 reduces TrkA activation and downstream signaling mostly in male DRG neurons. **A** Representative Western blot from lysates of male and female cultured TrkA-expressing DRG neurons from WT and *Ndfip2* cKO mice upon NGF stimulation (50 ng/ml) for the indicated time. Blots show activation and levels of TrkA, PLCγ, and ERK1/2 and deletion of Ndfip2 protein in *Ndfip2* cKO samples. Actin was used as loading control. **B** Quantification of TrkA levels. Note the reduced levels of TrkA in male, but not female, *Ndfip2* cKO. **C** Quantification of phosphorylated TrkA. **D** TrkA phosphorylation dynamics, relative to activation state at 5 min for each sample. **E** Quantification of phosphorylated PLCγ. **F** PLCγ activation dynamics similar as shown in panel D. **G** Quantification of phosphorylated ERK1/2. **H** ERK1/2 activation dynamics similar as shown in (**D**). For **B**–**H** (*n* = 5, 6 and *n* = 3-4 for males and females, respectively; values represented as mean ± SEM; WMann-Whitney test). **I** Representative Western blot of TrkA ubiquitination from male and female cultured DRG neurons from WT and Ndfip2 cKO mice. **J** Quantification of TrkA ubiquitination normalized with pTrkA and relative to control samples (*n* = 4 and 3 for males and females, respectively; mean ± SEM; *W*_Mann-Whitney_ test). **K** Representative Western blot showing cell surface proteins labeled with biotin (Surface, Sfc) and intracellular proteins (Intracellular, Int) upon Ndfip2 knockdown. **L** Quantification of TrkA levels relative to total TrkA in infected samples (*n* = 4; mean ± SEM; WMann-Whitney test). Note the increased amount of surface TrkA in Ndfip2 knockdown male neurons. **M** Representative Western blot of the different glycosylation states of TrkA upon co-expression with increasing levels of Ndfip2 and Nedd4l. **N** Quantification of fully glycosylated TrkA (140 kDa). **O** partially glycosylated TrkA (110 kDa), and **P** non-glycosylated TrkA (80 kDa) as for **J**. For **N**–**P** (*n* = 4; mean ± SEM; Dunn’s test for pairwise multiple comparisons).

To explore the role of Ndfip2 in TrkA signaling and dynamics, we cultured DRG neurons from E13.5 *Ndfip2* cKO and control mouse embryos, sorted by both sex and genotype. We observed a reduction of TrkA protein levels in cKO cultures for both sexes (Fig. 5A), but this reduction reached statistical significance only in males (Fig. 5B). Upon NGF stimulation, TrkA phosphorylation was significantly reduced in male Ndfip2 cKO neurons at 5 min, whereas female cultures maintained TrkA phosphorylation levels comparable to controls (Fig. 5C). Interestingly, 60 min after NGF treatment, TrkA phosphorylation was elevated in female cKO neurons (Fig. 5C). While females showed a slower decay than males in TrkA phosphorylation, Ndfip2 deletion did not alter this dynamic (Fig. 5D). Downstream signaling was also differentially affected. In males, PLCγ phosphorylation was significantly decreased at all time points (Fig. 5E) without altering its temporal dynamics (Fig. 5F). Nevertheless, ERK1/2 phosphorylation was significantly reduced only at early NGF exposure (5 min) in male and female Ndfip2 cKO neurons, while the overall activation profile remained unchanged (Fig. 5H). Thus, Ndfip2 regulates TrkA protein levels and NGF-mediated signaling more prominently in cultured male DRG neurons.

Because Ndfip2 is a protein associated with the function of the Nedd4-family of E3 ubiquitin ligases, we examined NGF-mediated TrkA ubiquitination in cKO cultures. After NGF stimulation for 15 min, ubiquitinated TrkA levels in *Ndfip2* cKO cultures were comparable to controls (Fig. 5I, J), indicating that Ndfip2 does not contribute to NGF-mediated TrkA ubiquitination.

### Ndfip2 modulates TrkA glycosylation and location at the plasma membrane

Considering Ndfip2 localization in ER and GA and TrkA reduced levels upon Ndfip2 depletion, we explored the TrkA biosynthetic pathway. First, we assessed basal TrkA cell surface localization in cultured, sex sorted DRG neurons following Ndfip2 knockdown. Interestingly, while intracellular TrkA levels were reduced in male shNdfip2 neurons (*ĝ*_Hedges_ = 1.54, *p* = 0.088), cell membrane-associated TrkA significantly increased by up to 40% (*ĝ*_Hedges_ = −2.38, *p* = 0.03), relative to total TrkA levels (Fig. 5K, L). No differences were observed in female neurons knocked down for Ndfip2. These results indicate that Ndfip2 modulates TrkA cell surface localization in male DRG neurons. Since TrkA needs to be fully glycosylated to be inserted into the plasma membrane, we took advantage of the expression of TrkA in HEK293FT cells, where the three bands corresponding to distinct glycosylation states of TrkA can be detected [31]. Ndfip2 expression led to a marked reduction in both the fully glycosylated (140 kDa) and partially glycosylated (110 kDa) forms of TrkA—by approximately 90% and 80%, respectively—compared with controls (Fig. 5M–O). In contrast, the non-glycosylated TrkA form (80 kDa) showed a 100% increase upon Ndfip2 expression (Fig. 5P). Co-expression of Nedd4-2 reduced Ndfip2 levels and normalized the accumulation of non-glycosylated TrkA but failed to restore mature or partially glycosylated forms (Fig. 5M–P). Additionally, increasing the amount of transfected TrkA plasmid in the presence of Ndfip2 did not rescue the mature TrkA levels (Fig. 5N). Together, these results suggest that Ndfip2 regulates TrkA glycosylation and maturation, leading to the accumulation of its immature, non-glycosylated form in HEK293FT cells.

## Discussion

The involvement of the NGF/TrkA axis in nociception is well documented and remains a major focus in pain research and drug development [32–34]. The ubiquitination of TrkA receptor through Nedd4-2 is key in the modulation of downstream signaling and pain [8–10, 35]. Nedd4-2 activity can be regulated by adaptor proteins such as the Ndfips, which influence ligase activation, subcellular localization, and substrate targeting [18, 25–27, 36]. Interestingly, transcriptomic analysis of DRGs from a mouse model of TrkA with reduced ubiquitination under painful conditions revealed that Ndfip2 expression is downstream of NGF/TrkA signaling [3]. These data suggested a potential feedback mechanism in which NGF/TrkA signaling may modulate its own activity through regulation of Ndfip2. Here, we demonstrate that Ndfip2 is expressed in adult DRG neurons, interacts with TrkA and regulates mechanical pain. Mechanistically, Ndfip2 deletion in TrkA-expressing cells modulates TrkA levels and activation.

Ndfip2 is expressed in several tissues, including the brain [20] and developing spinal cord [16], but its presence in adult DRG neurons had not been previously characterized. We demonstrate that Ndfip2 is expressed in DRG neurons, including TrkA-positive populations, and localizes to the ER and GA (Fig. 1B, C), consistent with its known transmembrane structure [12, 14]. Notably, although it has been reported that Ndfip2 controls the divalent metal ion transporter DMT1 in the liver of specifically female mice [25], we report a previously unrecognized sexual dimorphism in Ndfip2 expression, with higher protein and mRNA levels in male DRGs. The mechanism responsible for Ndfip2 sexual differences still requires further investigation.

TrkA interacts with Ndfip2 in sensory neurons, but contrary to previous studies in other cell types [15, 20, 21], we did not detect an endogenous interaction between Ndfip2 and Nedd4-2 (Fig. 2E). This may account for the absence of changes in TrkA ubiquitination upon NGF stimulation in Ndfip2 depleted neurons (Fig. 5I). Conversely, we observed an endogenous interaction between Ndfip1 and Nedd4-2, in agreement with previous reports [15, 20, 37], but no association between Ndfip1 and TrkA was detected in DRG neurons, despite earlier findings in HEK293 cells [23]. These differences may be explained within the cell context used for the different studies.

In adulthood, TrkA-expressing neurons mediate responses to heat, cold, and inflammatory pain [10, 35, 38]. To our surprise, *Ndfip2* cKO mice did not display differences in thermal nociception or in the inflammatory phase of the formalin test. In contrast, both male and female *Ndfip2* cKO mice exhibited lower mechanical pain, which is mediated by TrkA-dependent high-threshold mechanoreceptors [28]. As expected, non-noxious touch sensitivity (low-threshold, TrkB/C- and Ret-dependent neurons [28]) was unaffected in *Ndfip2* cKO mice. The different results produced by the two different von Frey methods used may indicate the activation of different neuronal populations in each case. While manual von Frey testing has been suggested to recruit low-threshold mechanoreceptors, electronic von Frey testing is thought to activate high-threshold mechanonociceptors more effectively [39]. This neurophysiological specificity has not yet been completely explored.

We tried to explain mechanistically the phenotypes observed in *Ndfip2* cKO mice addressing different questions. First, Ndfip2 deletion does not change total neuronal density in adult DRGs, discarding a general effect on cell death and survival (Fig. 4A, B). Second, Ndfip2 deletion led to reduced TrkA protein levels and decreased phosphorylation of TrkA and its downstream signaling effectors, mainly in males (Fig. 5A, B). Third, like many membrane receptors, TrkA undergoes glycosylation prior to plasma membrane insertion [31]. TrkA cell surface distribution following Ndfip2 knockdown upon NGF starvation increased in males (Fig. 5K, L), but Ndfip2 expression caused a marked reduction in glycosylated (mature) TrkA forms and accumulation of the non-glycosylated species (Fig. 5M–P). A similar modulatory role of Ndfip2 on the glycosylation of membrane proteins has been described previously [15]. This study shows that Ndfip2 recruits Nedd4-2 to MVBs, which impairs the degradation of mature hERG proteins before they reach the plasma membrane. In the case of TrkA, the E3 ubiquitin ligase that controls this biosynthetic process remains unknown. Combined with the localization of Ndfip2 to the ER and Golgi apparatus, our findings support a role for Ndfip2 in regulating TrkA maturation and trafficking to the plasma membrane. This function appears to be independent of Nedd4-2, as no interaction between these proteins was detected, although we cannot exclude the involvement of other Nedd4 family ubiquitin ligases. Altogether, these results support the notion that Ndfip2 may exert both Nedd4-dependent and Nedd4-independent functions. Whether this latter mechanism reflects a more general role of Ndfip2 in regulating the glycosylation and trafficking of transmembrane proteins remains to be determined.

There are several limitations of our study, such as we have focused on TrkA-positive sensory neurons, but there are other neurons in the DRGs that express Ndfip2 (Fig. 2G). In addition, although we studied primarily Ndfip2 on TrkA-expressing sensory neurons, other cell types that express TrkA—such as mast cells [35]—would also undergo Ndfip2 deletion in our cKO model, potentially influencing nociceptive phenotypes. Future research should investigate the role of Ndfip2 in other DRG neurons and in non-neuronal TrkA-expressing cells to fully understand its contribution to nociception and pain signaling.

In summary, this study identifies Ndfip2 as a key regulator of the maturation and post-translational modification of the TrkA receptor, a major component of nociceptive signaling. By linking early secretory trafficking mechanisms to sex-dependent pain behaviors, our findings reveal a novel mechanistic framework to explore Ndfip2’s broader physiological roles across sensory modalities and pain-related disorders.

## Materials and methods

### Mouse strains

All the experimental procedures involving animal subjects were performed following the European Community guidelines (63/2010), the Spanish Royal Decree 53/2013 and the Order ECC/566/2015, of 20th March. Protocols were previously approved by the Bioethics Committee of the University of Salamanca. Mice were housed in SPF Animal Facility of the University of Salamanca with a maximum of 5 animals per cage. They were fed *ad libitum* in a 12-h-light/dark cycle, constant temperature of 20–22 °C and a relative humidity of 55–65%. The strategy used to get the *Ndfip2* cKO in TrkA expressing cells is shown in Fig. 2A. In brief, we purchased a full reporter KO mouse for *Ndfip2* from the EMMA repository (EM:05045) and eliminated the reporter cassette by crossing it with animals expressing the Flp recombinase. We obtained a mouse with exon 3 flanked by loxP sites expressing normal levels of Ndfip2. These animals were crossed with others expressing the Cre recombinase under the *Ntrk1* promoter. The resulting mouse has one copy of the Cre recombinase and both alleles of *Ndfip2* floxed. Littermates with one or both alleles floxed and without Cre were used as controls.

### Mouse genotyping

To genotype mice, different PCR strategies were designed, using genomic DNA isolated from the remains of ear biopsies. First, Ndfip2 alleles were amplified using a triplex strategy: Ndfip2a: 5′-CAGCATTGATGCATGGTCAGC-3′, Ndfip2b: 5′-GATTACAAATGCTCCTGCAGG-3′ and Ndfip2c: 5′-CAACGGGTTCTTCTGTTAGTCC-3′. In addition, Ndfip2d primer 5′-TGGACATGGATGATGGCCAAA-3′ was used in combination with Ndfip2a to confirm recombination in mouse DRGs. Second, the Cre recombinase gene under the TrkA promoter was also detected working as a triplex, with a common forward primer 5′-CACCCAGTTACCTGGACGTTCTGGG-3′, a reverse primer for the WT allele 5′-GGGACCAAAATGGAAATTGATTCCAG-3′ and a reverse for the allele with the Cre 5′-AAGGAAAACCACGTCCCCGTGGTTC-3′. Third, for in vitro experiments pups were genotyped for sex determination, as described [40].

### HEK293FT cell culture

HEK293FT cells were grown in Dulbecco’s Modified Eagle Medium (DMEM) (Lonza, Switzerland) medium supplemented with 10% heat-inactivated bovine serum, 1% nonessential amino acids (Thermo Fisher Scientific), and 100 U/mL penicillin/streptomycin (Thermo Fisher Scientific).

### Primary TrkA-expressing DRG neuronal cultures

DRG were obtained from E15.5 rat and E13.5 mouse embryos, as previously described [38]. Rat embryos were pooled and processed together, while each mouse pup was processed individually for further genotyping and sorting. DRGs were enzymatically digested in Leibovitz-15 medium with 0.25% Trypsin/EDTA at 37 °C for 45 min with gentle agitation. Cells were centrifuged at 200 × *g* for 6 min and the pellet was resuspended in plating medium [MEM (Thermo Fisher Scientific, Spain), 10% FBS, 0.4% glucose, 2 mM L-glutamine, 100 U/mL penicillin/streptomycin (Thermo Fisher Scientific)] pipetting up and down until a single-cell suspension was obtained. Cells were plated on growth factor-reduced Matrigel (BD Biosciences, NY) coated wells with plating medium supplemented with 50 ng/ml NGF. The next day, the medium was replaced with Neurobasal (Thermo Fisher Scientific) supplemented with NS21, 0.4% glucose, 2 mM L-glutamine, NGF (25 ng/mL), and 5FU/U [fluorodeoxyuridine (2.44 μg/mL) and uridine (2.44 μg/mL)].

For Ndfip2 knockdown, neurons were infected at DIV 5 with lentivirus to modulate Ndfip2 levels for another 5 days. Infection was monitored using GFP expression. For signaling experiments, neurons were starved of NGF for 5 at DIV 10–12, and subsequently stimulated with 50 ng/ml NGF for 0, 5, 15, and 60 min. Samples were collected with Laemmli buffer for western blot.

### HEK293FT cells transient transfection

For transient transfection, HEK293FT cells were plated in Poly-D-lysine-coated plates. The day after, a ratio of 3:1 µg of polyethylenimine (PEI, Sigma-Aldrich #408727) per μg of plasmid DNA was diluted in OptiMEM and mixed by vortexing. After 20 min of incubation at RT, the solution was added to the culture, the cells were incubated for 4–6 h at 37 °C, and the medium was changed. One or two days later, lysates were collected in Laemmli buffer for Western blot.

### Lentivirus production

Lentiviruses were generated by transfecting HEK293FT cells with 10 μg (1.36 pmol) of the desired plasmids and the helpers encoding the capsid (0.61 pmol pMD.2 G) and the enveloping machinery (0.55 pmol psPAX2) [41]. After 5 hours, the medium was changed to DMEM without antibiotics. Two days later, the medium containing the viruses was centrifuged at 500 × *g* for 5 min, and supernatant ultracentrifuged to purify lentiviruses. Pellet was resuspended in Neurobasal medium and stored at −80 °C until use. The infectivity ratio was estimated using HEK293FT cells and DRG neurons, using GFP expression to calculate the ratio between infected and non-infected cells over the number of cells in the well. For further experiments, neuronal cultures were infected at a ratio of 4 infection viruses per cell. The efficacy of the knockdown or the overexpression was assessed by Western blot. For a proper KD of Ndfip2 a combination of two shRNAs was used.

### Cell surface biotinylation assay

The protocol for cell surface protein isolation was adapted [42] from Ayon-Olivas and Wolf et al. using the Pierce Surface biotinylation kit (Thermo Scientific; 89881). Mouse embryo dissections were performed as described and each pup was coated individually and sorted by sex. From each embryo, 2–3 × 10^5^ DRG cells were plated on polyornithine and laminin treated coverslips per 24-well plate, and infected at DIV5 to knock down Ndfip2 levels. At DIV 10, neurons were starved of NGF for 5 h, washed with cold PBS + 1 mM CaCl_2_ + 0.5 mM MgCl_2_ at 4°C. Sulfo-NHS-SS-Biotin was prepared at 0.25 mg/ml in PBS and neurons were incubated for 30 min at 4 °C. Excess biotin was quenched with 0.1 M glycine and washed again with cold PBS + 1 mM CaCl_2_ + 0.5 mM MgCl_2_. Neurons were lysed with a lysis buffer for 45 min and centrifuged. The biotin-labeled proteins were pulled down with NeutroAvidin-agarose, washed and isolated by centrifugation. The supernatant was also collected for analysis as the “*flowthrough*”. Samples were prepared with a 2× Laemmli buffer for SDS-PAGE and Western blotting.

### Western blot

Western blot was performed and quantified as described [43]. Protein samples were separated by SDS-PAGE and blotted using specific antibodies. HRP-conjugated secondary antibodies were used to detect the primary antibodies. The MicroChemi development system was used for image acquisition and band intensity quantification was performed using FIJI and RStudio. Multiple images were used to establish a linear range for each protein and the slope was used for comparison. Full length uncropped original western blots used in the manuscript figures are included in Supplementary Data.

### DRG cell counting and immunofluorescence quantification

L3 DRG ganglia from 3 to 4-month-old adult male and female mice were fixed in 0.4% PFA and 2 mM MgCl2 in 0.1 M PB pH 7.4 for 24 h. Sections (10 μm) from control and Ndfip2 cKO were mounted on the same slide for comparison. The IF protocol was performed as described [37]. Specific controls were performed by removing primary antibodies, and any non-specific staining was detected. Confocal images were captured using a Leica Stellaris microscope with an objective HC PL APO CS2 20X/0.75 DRY with a resolution of 1.86 pixels/μm, and subsequently analyzed using FIJI. Individual neurons in the images were segmented using Cellpose 2.0 (*cyto* pre-trained model, customized via manual correction and iterative training) [44, 45]. Background-subtracted mean fluorescence intensity and cell counting were performed using custom FIJI macros (available at 10.5281/zenodo.15520743). The number of positive cells per image was normalized to tissue area to obtain cell density per mm^²^.

For co-localization analysis, a custom FIJI macro was used (10.5281/zenodo.15530082). It performs iterative deconvolution, applies intensity thresholds, and measures the individual and overlapping signal areas in pixel units within a manually defined region of interest (ROI). Values are interpreted as relative proportions.

### Quantitative RT-qPCR analysis

Total RNA was isolated from control (*Ndfip2*^*f/f*^*;TrkA*^*+/+*^) or Ndfip2 cKO (*Ndfip2*^*f/f*^*;TrkA*^*Cre/+*^) 2-month-old mouse DRGs using TRIzol reagent (Invitrogen). cDNA was reverse transcribed using Superscript II reverse transcriptase and random primers. cDNA sample concentrations were determined by measuring absorbance at 260 nm using a NanoDrop 2000C spectrophotometer (Thermo Scientific). The SYBR-Green was included in a 2× Master Mix from Applied Biosystems (SYBR Green dye, dNTPs, Passive, ROX, AmpliTaq Gold DNA polymerase). The final volume of each reaction was 20 μl: 10 μl of Master Mix, 0.8 μl (16 pmols) of each oligonucleotide, 7.4 μl of distilled water and 1 μl of cDNA at a concentration of 100 ng/μl.

### Behavioral experiments

Behavioral experiments were adapted from previously described [38]. Experiments were performed blind to genotype to avoid bias.

#### Open field

After 2 days of habituation in individual cages for 30 min, animals were placed in a white arena (50 × 40 × 25 cm) with a recording camera on top. Total distance, mean speed and time spent in the center of the field were analyzed by the ANY MAZE software.

#### Electronic von-Frey test

Previously habituated mice were placed on a grid surface. The sensitivity to potentially harmful mechanical stimuli was measured using the Ugo Basile analgesimeter, setting an increasing force ramp (5 g in 10 s, cutoff of 15 s). Three measures for each paw were taken with a 10 min recovery period.

#### Up-down von frey test

Animals were habituated for 2 days in individual Plexiglas cubes on a grid surface, the calibrated monofilaments (Touch Test Kit, North Coast Medical Inc.) were applied, and 50% paw withdrawal threshold was calculated as described [46]. The test was repeated in each paw on two consecutive days.

#### Acetone drop evaporation test

Mice previously habituated to the grid surface were stimulated from below with a drop of acetone on the hind paw. The response was recorded for one minute and time spent in nocifensive behavior (biting, licking, flicking, and hiding the stimulated limb) was quantified. Three measures of each paw were taken with a 10 min recovery period.

#### Dry ice test

Mice were placed in Plexiglas cubicles, on a glass surface, and habituated for 2 days. A homogeneous pellet of dry ice in a 5 ml syringe was applied through the glass to the hind paw and withdrawal latency was measured by two experimenters independently. Three measures of each paw were taken with a 10 min recovery period.

#### Cold plate test

A glass flask was placed on an ice box and cooled down to a surface temperature of 7–9 °C. Animals were located three times with a 10 min recovery period between repeats and their response was scored by two independent experimenters. The first two times mice were removed after the first cooling sign (removal of forepaws, rearing or jumping) and the third time the response was recorded for one minute and the number of rears plus jumps was analyzed.

#### Hargreaves’ test

Mice were habituated for two days in individual Plexiglas cubes on a glass surface. The Ugo Basile Plantar Test for Thermal Stimulation apparatus was used, calibrated to stimulate at 125 mW/cm^2^ with a cut-off time of 25 s. The time taken for each mouse to remove its paw was automatically recorded. Each paw was measured three times, alternating right and left, with at least 5 min interval between measurements.

#### Tail flick test

Animals were habituated for two days to be covered with a cloth with their tail fully extended on the surface of the Ugo Basile Tail Flick apparatus, which was calibrated to stimulate at 125 mW/cm^2^ with a 25-s cut-off time. The stimulus was applied at 3 cm from the tip of the tail, and the time until the tail was removed was automatically recorded. At least three measurements were taken from each mouse, with at least 5 min of recovery between measurements.

#### Formalin test

Mice were injected subcutaneously using a 26-G needle in the right hind paw with 10 µl of a 5% formaldehyde solution in PBS, and then placed on a glass surface in Plexiglass boxes with enough space to allow animals to move. Their response was recorded for 55 min and the time spent in nocifensive behavior was quantified at 5-min intervals. Phase I was considered for the first 15 minutes, and phase II from 15 to 55 min.

### Quantification and statistical analysis

Experiments were conducted blinded to genotype, and data were grouped by sex and genotype. Statistical analyses were performed using RStudio, employing the rstatix and ggstatsplot packages [47, 48]. Graphical data representations were generated with ggplot2 [49], showing mean ± SEM. T-test, Welch’s t-test or Wilcoxon-Mann-Whitney test was used for two-sample comparisons based on data characteristics. Normality and homoscedasticity were verified with Shapiro-Wilk and Levene’s tests. Pairwise comparisons were performed using t-tests, with Welch’s correction when necessary and Hedges’ *g* for effect size estimation, presented with confidence intervals and sample size. Quantification of the effect size magnitude was performed using the thresholds defined in Cohen (1992) [50], i.e., |d| < 0.2 “negligible”, |d| < 0.5 “small”, |d| < 0.8 “medium”, otherwise “large”.

**Resource table**

Summary of reagents, deposited data and softaware and algorithms

**Table:** 

Reagent or resource	Source	Identifier
**Antibodies**		
Mouse anti-Actin (WB)	Sigma	Cat#A4700
Chicken anti-Calreticulin (IF)	Thermo	Cat#PA1-902A
Rabbit anti-ERK1/2 (WB)	Cell Signaling	Cat#9102
Rabbit anti-Flag (WB)	Sigma	Cat#F7425
Mouse anti-Gapdh (WB)	Sigma	Cat#G8795
Mouse anti-GFP (WB)	Clontech	Cat#632380
Mouse anti-GM130 (IF)	B.D.	Cat#610822
Rat anti-Lamp1 (IF)	Dev. Studies Hybridoma Bank	Cat#AB_528127
Rabbit anti-Myc (WB)	Santa Cruz Biotech.	Cat#sc-789
Rabbit anti-Ndfip1 (WB)	Dr. Sharad Kumar	Harvey et al. [11]
Rabbit anti-Ndfip2 (WB, IF)	Dr. Sharad Kumar	Konstas et al. [21]
Rabbit anti-Nedd4-2 (WB)	Dr. Juan Carlos Arévalo	Arevalo et al. [8]
Mouse anti-NeuN (IF)	Milipore	Cat#MAB377
Rabbit anti-panCadherin (WB)	Invitrogen	Cat#71-7100
Rabbit anti-panTrk (203) (WB, IP)	Dr. Dionisio Martín Zanca	Martín-Zanca et al. [31]
Mouse anti-panTrk (B3) (WB)	Santa Cruz Biotech.	Cat#sc-7268
Mouse anti-pERK1/2 (WB)	Cell Signaling	Cat#9106
Mouse anti-PLCγ (WB)	Santa Cruz Biotech.	Cat#sc72-90
Rabbit anti-pPLCγ (WB)	Cell Siganling	Cat#2821
Rabbit anti-pTrk (Y490) (WB)	Cell Signaling	Cat#9141S
Mouse anti-TGN38 (IF)	Affinity Bioreagents	Cat#MA3-063
Mouse anti-TrkA (WB, IF)	R&D Systems	Cat#MAB1056
Rabbit anti-TrkA (RTA) (WB)	Dr. Louis Reichardt	Clary et al. [51]
Mouse anti-Ubiquitin (P4D1) (WB)	Santa Cruz Biotech.	Cat#sc-8017
Goat anti-Chicken Alexa Fluor 594 (IF)	Thermo	Cat#A-11042
Goat anti-Mouse Alexa Fluor 488 (IF)	Invitrogen	Cat#A11001
Goat anti-Mouse Alexa Fluor 555 (IF)	Invitrogen	Cat#A21424
Goat anti-Mouse HRP (WB)	Jackson Immunoresearch	Cat#AB_10015289
Goat anti-Rabbit Alexa Fluor 488 (IF)	Invitrogen	Cat#A11008
Goat anti-Rabbit Alexa Fluor 555 (IF)	Invitrogen	Cat#A21429
Goat anti-Rabbit HRP (WB)	Jackson Immunoresearch	Cat#AB_2313567
Donkey anti-Rat Cy3 (IF)	Jackson Immunoresearch	Cat#AB_2340667
**Bacterial and virus strains**		
Lenti pLVTHM-sh*mouse*Ndfip2#1	This paper	N/A
Lenti pLVTHM-sh*mouse*Ndfip2#2	This paper	N/A
Lenti pLVTHM-shControl	This paper	N/A
**Chemicals, peptides, and recombinant proteins**		
Human recombinant NGF	Alomone labs	Cat#N-100
NeuroTrace 530/615 Red Nissl Stain	Thermo Fisher Scientific	Cat#N21482
Polyethylenimine (PEI)	Sigma-Aldrich	Cat#408727
Matrigel (reduced growth factor)	BD Biosciences	Cat#356231
Poly-D-lysine	Sigma-Aldrich	Cat#P7280
Fluorodeoxyuridine (5FU)	Sigma-Aldrich	Cat#F0503
Uridine	Sigma-Aldrich	Cat#U3003
**Critical commercial assays**		
Pierce cell surface protein isolation kit	Thermo Scientific	Cat#89881
**Deposited data**		
FIJI Macro for Cell Fluorescence Quantification in Immunofluorescence Images	Zenodo	10.5281/zenodo.15520743
FIJI Macro for Colocalisation Analysis using Deconvolution	Zenodo	10.5281/zenodo.15530082
**Experimental models: Cell lines**		
HEK293FT	Dr. Moses Chao	N/A
**Experimental models: Organisms/strains**		
Ndfip2 reporter KO: C57BL/6N-Ndfip2^tm1a(EUCOMM)Hmgu^/Ieg	EMMA	EM05045
Ndfip2 cKO	This paper	N/A
TrkA^Cre^: B6;129S4-Ntrk1^tm1(cre)Lfr^Mmucd	MMRRC	015500-UCD
Flp deleter	Dr. Manuel A. Sánchez-Martín	Kranz et al. [52]
**Oligonucleotides**		
sh*mouse*Ndfip2#1: 5′-CGGGATGACTTCAGTGATG-3′	This paper	N/A
sh*mouse*Ndfip2#2: 5′-TACCATCGCTGGAAGATACGG-3′	This paper	N/A
shControl (*Euglena gracilis*): 5′-GCGCGCTTTGTAGGATTCG-3′	Arevalo et al. [8]	N/A
Ndfip2a: 5′-CAGCATTGATGCATGGTCAGC-3′	This paper	N/A
Ndfip2b: 5′-GATTACAAATGCTCCTGCAGG-3′	This paper	N/A
Ndfip2c: 5′-CAACGGGTTCTTCTGTTAGTCC-3′	This paper	N/A
Ndfip2d: 5′-TGGACATGGATGATGGCCAAA-3′	This paper	N/A
TrkA-FWD: 5′-CACCCAGTTACCTGGACGTTCTGGG-3′	Sánchez-Sánchez et al. [38]	N/A
TrkA-REV-WT: 5′-GGGACCAAAATGGAAATTGATTCCAG-3′	Sánchez-Sánchez et al. [38]	N/A
TrkA-REV-Cre: 5′-AAGGAAAACCACGTCCCCGTGGTTC-3′	Sánchez-Sánchez et al. [38]	N/A
**Recombinant DNA**		
pLVTHM	Addgene	Cat#12247
psPAX2	Addgene	Cat#12260
pMD2.G	Addgene	Cat#12259
pcDNA3-rTrkA	Dr. Dionisio Martín-Zanca	Arevalo et al. [53]
pCDNA3-rTrkB	Dr. Dionisio Martín-Zanca	N/A
pcDNA3-rTrkC	Dr. Dionisio Martín-Zanca	N/A
mNdfip2-Flag-pcDNA3	Dr. Sharad Kumar	Konstas et al. [21]
pEGFP-Nedd4-2	Dr. Moses Chao	Arévalo et al. [8]
**Software and algorithms**		
R/RStudio	R Foundation	https://www.r-project.org/
Cellpose2.0	Stringer and Pachitariu, [44]	https://www.cellpose.org
FIJI	Schindelin et al. [45]	https://fiji.sc
ANY-maze	Stoelting Co.	RRID: SCR_014289

## Supplementary information

Supplementary material

## Data Availability

Material and data will be provided upon reasonable request (arevalojc@usal.es) and at 10.5281/zenodo.15520743, 10.5281/zenodo.15530082 and https://pubmed.ncbi.nlm.nih.gov/37323629/.
