# Supplementary material for: Ndfip2 in TrkA-expressing sensory neurons regulates noxious mechanosensation through control of TrkA signaling and protein levels
Source: Cell Death Dis. 2026 Mar 31;17(1):437. doi: 10.1038/s41419-026-08670-9 (PMC13158297; doi:10.1038/s41419-026-08670-9)

**
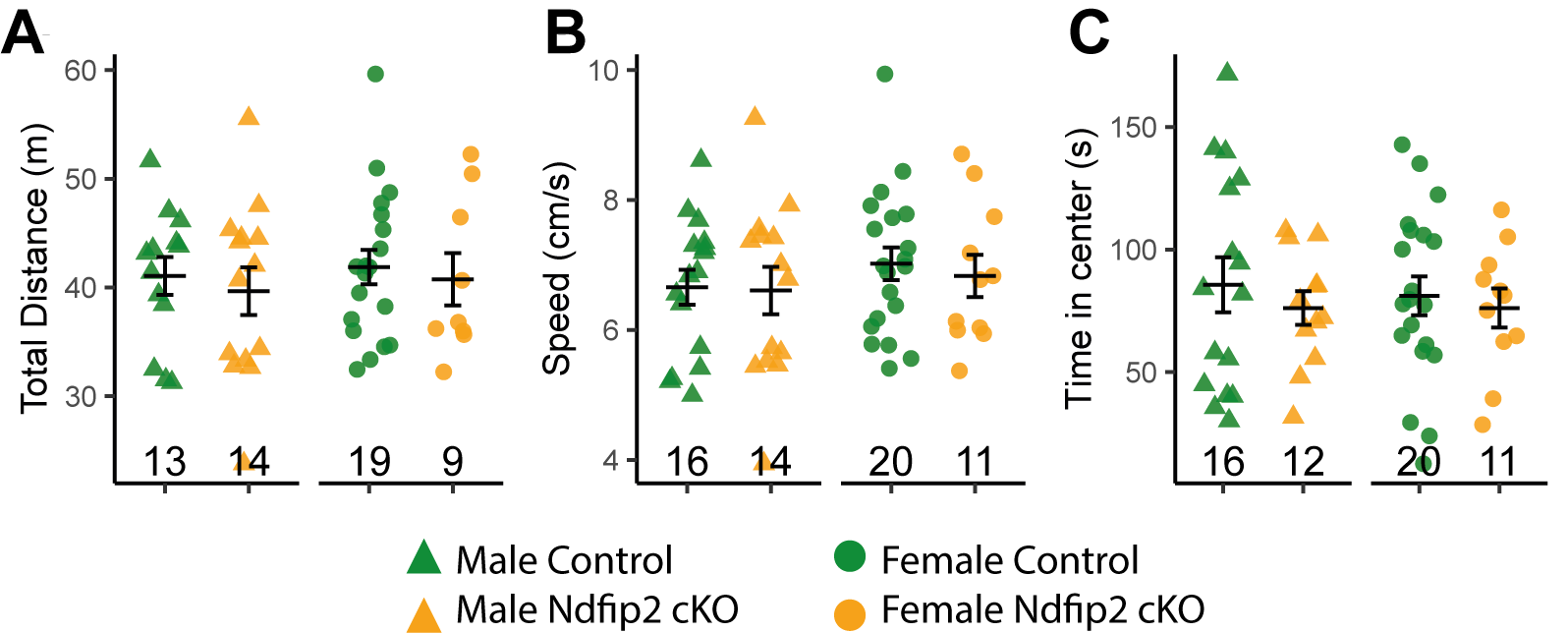
**

**Figure S1. *Ndfip2* cKO mice behave similarly to controls in the open field test. A)** Total distance and **B)** mean speed are shown to test general locomotion. **C)** Time spent in the center of the field as a measure of stress/anxiety. The number of animals tested for each group is shown (mean ± SEM; t-test, with Welch correction, where appropriate).

**Supplementary Data 1: Statistical analysis of behavioural experiments.** Statistical analyses were performed using the rstatix package in R. Outlier values were removed following appropriate testing. Data normality and homogeneity of variances were assessed using the Shapiro–Wilk and Levene’s tests, respectively. A two-way ANOVA was conducted with Sex and Genotype as between-subject factors. Pairwise comparisons were performed using t-tests, with Welch’s correction applied when necessary. Effect sizes were calculated using Hedges’ g and are reported with corresponding confidence intervals and sample sizes.

**Electronic von Frey**

Two-way ANOVA

| Effect | DFn | DFd | F | *p* | ges |
| --- | --- | --- | --- | --- | --- |
| Genotype | 1 | 35 | 10.42 | 0.003 | 0.23 |
| Sex | 1 | 35 | 6.18 | 0.018 | 0.15 |
| Genotype:Sex | 1 | 35 | 0.03 | 0.86 | 0.00 |

♂ Ctrl vs Ndfip2cKO: *t*_Student_(15) = -2.31, *p* = 0.035, *ĝ*_Hedges_ = -1.01, CI_95%_ [-1.99, -0.01], *n*_obs_ = 17

♀ Ctrl vs Ndfip2cKO: *t*_Student_(20) = -2.26, *p* = 0.035, *ĝ*_Hedges_ = -0.98, CI_95%_ [-1.84, -0.09], *n*_obs_ = 22

Ctrl ♂ vs ♀: *t*_Student_(21) = 2.20, *p* = 0.02, *ĝ*_Hedges_ = 0.04, CI_95%_ [0.14, 1.76], *n*_obs_ = 23

Ndfip2cKO ♂ vs ♀: *t*_Student_(14) = 1.32, *p* = 0.21, *ĝ*_Hedges_ = 0.62, CI_95%_ [-0.34, 1.56], *n*_obs_ = 16

**Up-Down von Frey**

Two-way ANOVA

| Effect | DFn | DFd | F | *p* | ges |
| --- | --- | --- | --- | --- | --- |
| Genotype | 1 | 35 | 1.90 | 0.18 | 0.05 |
| Sex | 1 | 35 | 3.03 | 0.09 | 0.08 |
| Genotype:Sex | 1 | 35 | 0.01 | 0.92 | 0.00 |

♂ Ctrl vs Ndfip2cKO: *t*_Student_(17) = 0.94, *p* = 0.36, *ĝ*_Hedges_ = 0.43, CI_95%_ [-0.43, 1.28], *n*_obs_ = 19

♀ Ctrl vs Ndfip2cKO: *t*_Student_(18) = 1.02, *p* = 0.32, *ĝ*_Hedges_ = 0.40, CI_95%_ [-0.51, 1.30], *n*_obs_ = 20

Ctrl ♂ vs ♀: *t*_Student_(22) = -1.43, *p* = 0.17, *ĝ*_Hedges_ = -0.54, CI_95%_ [-1.34, 0.27], *n*_obs_ = 24

Ndfip2cKO ♂ vs ♀: *t*_Student_(13) = -1.00, *p* = 0.34, *ĝ*_Hedges_ = -0.48, CI_95%_ [-1.44, 0.51], *n*_obs_ = 15

**Acetone drop test**

Two-way ANOVA with heteroscedasticity correction

| Effect | DFn | DFd | F | *p* |
| --- | --- | --- | --- | --- |
| Genotype | 1 | 56 | 3.85 | 0.055 |
| Sex | 1 | 56 | 9.14 | 0.004 |
| Genotype:Sex | 1 | 56 | 0.03 | 0.87 |

♂ Ctrl vs Ndfip2cKO: *t*_Welch_(22.09) = 1.81, *p* = 0.08, *ĝ*_Hedges_ = 0.70, CI_95%_ [-0.09, 1.48], *n*_obs_ = 25

♀ Ctrl vs Ndfip2cKO: *t*_Welch_(30.29) = 0.96, *p* = 0.35, *ĝ*_Hedges_ = 0.32, CI_95%_ [-0.34, 0.97], *n*_obs_ = 35

Ctrl ♂ vs ♀: *t*_Welch_(33.50) = 2.10, *p* = 0.04, *ĝ*_Hedges_ = 0.62, CI_95%_ [-0.02, 1.28], *n*_obs_ = 37

Ndfip2cKO ♂ vs ♀: *t*_Welch_(19.31) = 2.33, *p* = 0.03, *ĝ*_Hedges_ = 0.91, CI_95%_ [0.08, 1.71], *n*_obs_ = 23

**Dry ice test**

Two-way ANOVA

| Effect | DFn | DFd | F | *p* | ges |
| --- | --- | --- | --- | --- | --- |
| Genotype | 1 | 32 | 0.61 | 0.44 | 0.02 |
| Sex | 1 | 32 | 0.02 | 0.88 | 0.00 |
| Genotype:Sex | 1 | 32 | 1.50 | 0.23 | 0.05 |

♂ Ctrl vs Ndfip2cKO: *t*_Student_(14) = 1.56, *p* = 0.14, *ĝ*_Hedges_ = 0.72, CI_95%_ [-0.25, 1.66], *n*_obs_ = 16

♀ Ctrl vs Ndfip2cKO: *t*_Student_(19) = -0.23, *p* = 0.82, *ĝ*_Hedges_ = -0.10, CI_95%_ [-0.94, 0.75], *n*_obs_ = 20

Ctrl ♂ vs ♀: *t*_Student_(17) = -0.91, *p* = 0.37, *ĝ*_Hedges_ = -0.39, CI_95%_ [-1.27, 0.50], *n*_obs_ = 19

Ndfip2cKO ♂ vs ♀: *t*_Student_(15) = 0.83, *p* = 0.42, *ĝ*_Hedges_ = 0.38, CI_95%_ [-0.51, 1.27], *n*_obs_ = 17

**Cold plate - First sign**

Two-way ANOVA with heteroscedasticity correction

| Effect | DFn | DFd | F | *p* |
| --- | --- | --- | --- | --- |
| Genotype | 1 | 32 | 0.16 | 0.69 |
| Sex | 1 | 32 | 6.02 | 0.02 |
| Genotype:Sex | 1 | 32 | 0.78 | 0.38 |

♂ Ctrl vs Ndfip2cKO: *t*_Welch_(14.88) = 0.13, *p* = 0.90, *ĝ*_Hedges_ = 0.06, CI_95%_ [-0.84, 0.96], *n*_obs_ = 17

♀ Ctrl vs Ndfip2cKO: *t*_Welch_(9.33) = -1.02, *p* = 0.33, *ĝ*_Hedges_ = -0.44, CI_95%_ [-1.30, 0.44], *n*_obs_ = 19

Ctrl ♂ vs ♀: *t*_Welch_(12.21) = 1.92, *p* = 0.08, *ĝ*_Hedges_ = 0.87, CI_95%_ [-0.10, 1.81], *n*_obs_ = 18

Ndfip2cKO ♂ vs ♀: *t*_Welch_(11.06) = 2.00, *p* = 0.07, *ĝ*_Hedges_ = 0.88, CI_95%_ [-0.07, 1.79], *n*_obs_ = 18

**Cold plate - Number of scapes**

Two-way ANOVA with heteroscedasticity correction

| Effect | DFn | DFd | F | *p* |
| --- | --- | --- | --- | --- |
| Genotype | 1 | 32 | 0.45 | 0.51 |
| Sex | 1 | 32 | 0.43 | 0.52 |
| Genotype:Sex | 1 | 32 | 1.72 | 0.20 |

♂ Ctrl vs Ndfip2cKO: *t*_Welch_(14.96) = 1.29, *p* = 0.22, *ĝ*_Hedges_ = 0.59, CI_95%_ [-0.34, 1.51], *n*_obs_ = 17

♀ Ctrl vs Ndfip2cKO: *t*_Welch_(11.99) = -0.88, *p* = 0.40, *ĝ*_Hedges_ = -0.38, CI_95%_ [-1.25, 0.49], *n*_obs_ = 19

Ctrl ♂ vs ♀: *t*_Welch_(15.83) = -1.31, *p* = 0.21, *ĝ*_Hedges_ = -0.58, CI_95%_ [-1.46, 0.32], *n*_obs_ = 18

Ndfip2cKO ♂ vs ♀: *t*_Welch_(11.19) = 0.84, *p* = 0.42, *ĝ*_Hedges_ = 0.37, CI_95%_ [-0.51, 1.24], *n*_obs_ = 18

**Hargreaves’ test**

Two-way ANOVA

| Effect | DFn | DFd | F | *p* | ges |
| --- | --- | --- | --- | --- | --- |
| Genotype | 1 | 34 | 0.00 | 0.97 | 0.00 |
| Sex | 1 | 34 | 5.51 | 0.03 | 0.14 |
| Genotype:Sex | 1 | 34 | 0.36 | 0.55 | 0.01 |

♂ Ctrl vs Ndfip2cKO: *t*_Student_(16) = 0.42, *p* = 0.68, *ĝ*_Hedges_ = 0.18, CI_95%_ [-0.72, 1.06], *n*_obs_ = 18

♀ Ctrl vs Ndfip2cKO: *t*_Student_(18) = -0.43, *p* = 0.68, *ĝ*_Hedges_ = -0.20, CI_95%_ [-1.02, 0.62], *n*_obs_ = 20

Ctrl ♂ vs ♀: *t*_Student_(21) = -2.37, *p* = 0.03, *ĝ*_Hedges_ = -0.98, CI_95%_ [-1.80, -0.13], *n*_obs_ = 23

Ndfip2cKO ♂ vs ♀: *t*_Student_(13) = -0.92, *p* = 0.37, *ĝ*_Hedges_ = -0.45, CI_95%_ [-1.38, 0.50], *n*_obs_ = 15

**Tail flick**

Two-way ANOVA with heteroscedasticity correction

| Effect | DFn | DFd | F | *p* |
| --- | --- | --- | --- | --- |
| Genotype | 1 | 51 | 0.62 | 0.44 |
| Sex | 1 | 51 | 5.18 | 0.03 |
| Genotype:Sex | 1 | 51 | 0.04 | 0.85 |

♂ Ctrl vs Ndfip2cKO: *t*_Welch_(20.03) = -0.67, *p* = 0.51, *ĝ*_Hedges_ = -0.24, CI_95%_ [-0.92, 0.46], *n*_obs_ = 26

♀ Ctrl vs Ndfip2cKO: *t*_Welch_(13.79) = -0.50, *p* = 0.62, *ĝ*_Hedges_ = -0.19, CI_95%_ [-0.95, 0.57], *n*_obs_ = 29

Ctrl ♂ vs ♀: *t*_Welch_(31.96) = 1.93, *p* = 0.06, *ĝ*_Hedges_ = 0.65, CI_95%_ [-0.04, 1.31], *n*_obs_ = 34

Ndfip2cKO ♂ vs ♀: *t*_Welch_(10.67) = 1.38, *p* = 0.20, *ĝ*_Hedges_ = 0.55, CI_95%_ [-0.28, 1.34], *n*_obs_ = 21

**Formalin injection test**

**5 m:** ♂ Ctrl vs Ndfip2cKO: *t*_Welch_(10.5) = -0.76, *p* = 0.46, *ĝ*_Hedges_ = -0.39, CI_95%_ [-1.41, 0.64], *n*_obs_ =13

♀ Ctrl vs Ndfip2cKO: *t*_Welch_(8.86) = -1.45, *p* = 0.18, *ĝ*_Hedges_ = -0.75, CI_95%_ [1.81, 0.34], *n*_obs_ = 12

**10 m:** ♂ Ctrl vs Ndfip2cKO: *t*_Welch_(11) = -0.35, *p* = 0.73, *ĝ*_Hedges_ = -0.18, CI_95%_ [-1.19, 0.83], *n*_obs_ = 13

♀ Ctrl vs Ndfip2cKO: *t*_Welch_(9.77) = -3.26, *p* = 0.009, *ĝ*_Hedges_ = -1.66, CI_95%_ [-2.87, -0.40], *n*_obs_ = 12

**15 m:** ♂ Ctrl vs Ndfip2cKO: *t*_Welch_(9.56) = -1.49, *p* = 0.17, *ĝ*_Hedges_ = -0.77, CI_95%_ [-1.82, 0.32], *n*_obs_ = 13

♀ Ctrl vs Ndfip2cKO: *t*_Welch_(4.88) = -0.99, *p* = 0.37, *ĝ*_Hedges_ = -0.53, CI_95%_ [-1.60, 0.59], *n*_obs_ = 12

**20 m:** ♂ Ctrl vs Ndfip2cKO: *t*_Welch_(10.99) = 0.78, *p* = 0.45, *ĝ*_Hedges_ = 0.40, CI_95%_ [-0.63, 1.42], *n*_obs_ = 13

♀ Ctrl vs Ndfip2cKO: *t*_Welch_(4.8) = -0.24, *p* = 0.82, *ĝ*_Hedges_ = -0.13, CI_95%_ [-1.17, 0.93], *n*_obs_ = 12

**25 m:** ♂ Ctrl vs Ndfip2cKO: *t*_Welch_(10.14) = 0.57, *p* = 0.58, *ĝ*_Hedges_ = 0.30, CI_95%_ [-0.73, 1.31], *n*_obs_ = 13

♀ Ctrl vs Ndfip2cKO: *t*_Welch_(6.57) = -0.96, *p* = 0.37, *ĝ*_Hedges_ = -0.51, CI_95%_ [-1.57, 0.58], *n*_obs_ = 12

**30 m:** ♂ Ctrl vs Ndfip2cKO: *t*_Welch_(10.88) = 0.69, *p* = 0.51, *ĝ*_Hedges_ = 0.35, CI_95%_ [-0.67, 1.37], *n*_obs_ = 13

♀ Ctrl vs Ndfip2cKO: *t*_Welch_(9.92) = 0.34, *p* = 0.74, *ĝ*_Hedges_ = 0.17, CI_95%_ [-0.83, 1.16], *n*_obs_ = 12

**35 m:** ♂ Ctrl vs Ndfip2cKO: *t*_Welch_(9.99) = -0.04, *p* = 0.97, *ĝ*_Hedges_ = -0.02, CI_95%_ [-1.03, 0.99], *n*_obs_ = 13

♀ Ctrl vs Ndfip2cKO: *t*_Welch_(4.78) = -1.31, *p* = 0.25, *ĝ*_Hedges_ = -0.70, CI_95%_ [-1.80, 0.46], *n*_obs_ = 12

**40 m:** ♂ Ctrl vs Ndfip2cKO: *t*_Welch_(8.84) = -0.33, *p* = 0.75, *ĝ*_Hedges_ = -0.17, CI_95%_ [-1.17, 0.85], *n*_obs_ = 13

♀ Ctrl vs Ndfip2cKO: *t*_Welch_(6.15) = -0.65, *p* = 0.54, *ĝ*_Hedges_ = -0.34, CI_95%_ [-1.39, 0.73], *n*_obs_ = 12

**45 m:** ♂ Ctrl vs Ndfip2cKO: *t*_Welch_(9.77) = 0.04, *p* = 0.97, *ĝ*_Hedges_ = 0.02, CI_95%_ [-1.02, 1.06], *n*_obs_ = 12

♀ Ctrl vs Ndfip2cKO: *t*_Welch_(3.79) = -1.67, *p* = 0.17, *ĝ*_Hedges_ = -0.88, CI_95%_ [-2.03, 0.36], *n*_obs_ = 12

**50 m:** ♂ Ctrl vs Ndfip2cKO: *t*_Welch_(7.05) = -0.20, *p* =0.85 , *ĝ*_Hedges_ = -0.11, CI_95%_ [-1.21, 1.00], *n*_obs_ = 10

♀ Ctrl vs Ndfip2cKO: *t*_Welch_(7.7) = 0.95, *p* = 0.37, *ĝ*_Hedges_ = 0.50, CI_95%_ [-0.58, 1.55], *n*_obs_ = 12

**55 m:** ♂ Ctrl vs Ndfip2cKO: *t*_Welch_(7.73) = -0.11, *p* = 0.91, *ĝ*_Hedges_ = -0.06, CI_95%_ [-1.18, 1.05], *n*_obs_ = 10

♀ Ctrl vs Ndfip2cKO: *t*_Welch_(4.54) = 0.18, *p* = 0.87, *ĝ*_Hedges_ = 0.09, CI_95%_ [-0.96, 1.13], *n*_obs_ = 12

**Open field - Distance**

Two-way ANOVA

| Effect | DFn | DFd | F | *p* | ges |
| --- | --- | --- | --- | --- | --- |
| Genotype | 1 | 51 | 0.41 | 0.53 | 0.01 |
| Sex | 1 | 51 | 0.22 | 0.64 | 0.00 |
| Genotype:Sex | 1 | 51 | 0.01 | 0.95 | 0.00 |

♂ Ctrl vs Ndfip2cKO: *t*_Student_(25) = 0.50, *p* = 0.62, *ĝ*_Hedges_ = 0.19, CI_95%_ [-0.54, 0.91], *n*_obs_ = 27

♀ Ctrl vs Ndfip2cKO: *t*_Student_(26) = 0.40, *p* = 0.69, *ĝ*_Hedges_ = 0.15, CI_95%_ [-0.61, 0.91], *n*_obs_ = 28

Ctrl ♂ vs ♀: *t*_Student_(30) = 0.34, *p* = 0.74, *ĝ*_Hedges_ = 0.12, CI_95%_ [-0.56, 0.80], *n*_obs_ = 32

Ndfip2cKO ♂ vs ♀: *t*_Student_(21) = 0.33, *p* = 0.75, *ĝ*_Hedges_ = 0.14, CI_95%_ [-0.66, 0.93], *n*_obs_ = 23

**Open field - Speed**

Two-way ANOVA

| Effect | DFn | DFd | F | *p* | ges |
| --- | --- | --- | --- | --- | --- |
| Genotype | 1 | 57 | 0.15 | 0.70 | 0.00 |
| Sex | 1 | 57 | 1.03 | 0.31 | 0.02 |
| Genotype:Sex | 1 | 57 | 0.05 | 0.82 | 0.00 |

♂ Ctrl vs Ndfip2cKO: *t*_Student_(28) = 0.11, *p* = 0.91, *ĝ*_Hedges_ = 0.04, CI_95%_ [-0.66, 0.74], *n*_obs_ = 30

♀ Ctrl vs Ndfip2cKO: *t*_Student_(29) = 0.45, *p* = 0.65, *ĝ*_Hedges_ = 0.16, CI_95%_ [-0.54, 0.87], *n*_obs_ = 31

Ctrl ♂ vs ♀: *t*_Student_(34) = 0.98, *p* = 0.33, *ĝ*_Hedges_ = 0.32, CI_95%_ [-0.33, 0.96], *n*_obs_ = 36

Ndfip2cKO ♂ vs ♀: *t*_Student_(23) = 0.44, *p* = 0.66, *ĝ*_Hedges_ = 0.18, CI_95%_ [-0.58, 0.93], *n*_obs_ = 25

**Open Field - Time in centre**

Two-way ANOVA with heteroscedasticity correction

| Effect | DFn | DFd | F | *p* |
| --- | --- | --- | --- | --- |
| Genotype | 1 | 55 | 0.61 | 0.44 |
| Sex | 1 | 55 | 0.04 | 0.85 |
| Genotype:Sex | 1 | 55 | 0.06 | 0.80 |

♂ Ctrl vs Ndfip2cKO: *t*_Welch_(23.80) = 0.72, *p* = 0.48, *ĝ*_Hedges_ = 0.26, CI_95%_ [-0.44, 0.95], *n*_obs_ = 28

♀ Ctrl vs Ndfip2cKO: *t*_Welch_(26.25) = 0.44, *p* = 0.66, *ĝ*_Hedges_ = 0.15, CI_95%_ [-0.53, 0.84], *n*_obs_ = 31

Ctrl ♂ vs ♀: *t*_Welch_(28.25) = -0.33, *p* = 0.75, *ĝ*_Hedges_ = -0.11, CI_95%_ [-0.76, 0.54], *n*_obs_ = 36

Ndfip2cKO ♂ vs ♀: *t*_Welch_(20.26) = 2e-03, *p* = 1.00, *ĝ*_Hedges_ = 8.2e-04, CI_95%_ [-0.79, 0.79], *n*_obs_ = 23

**Supplementary Data 2: Original western blots.** Full length uncropped original western blots used in the manuscript are shown, sorted by figure and panel.

**Figure 1**

**D**

**
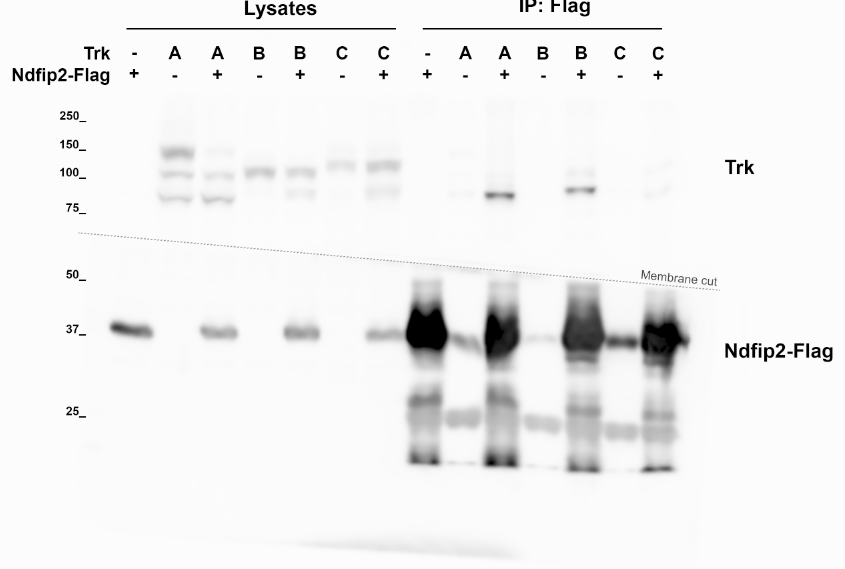
**

**
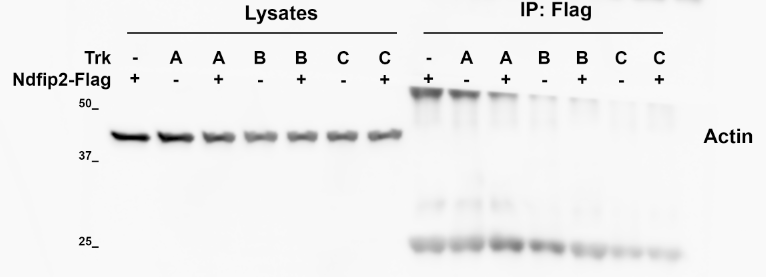
**

**E**


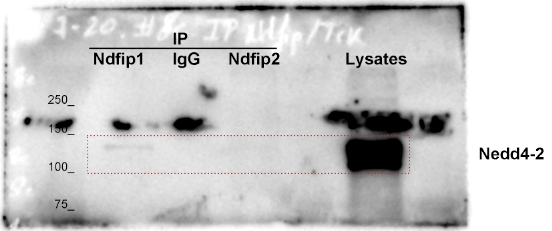

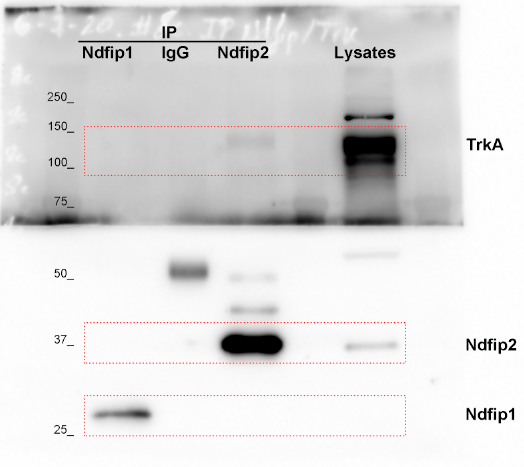


**Figure 2**

**F**

**
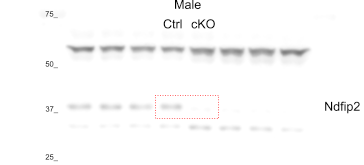

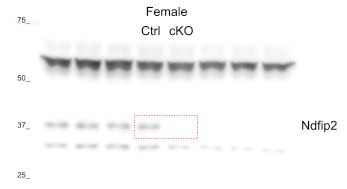
**

**
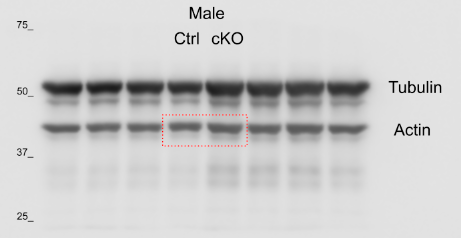

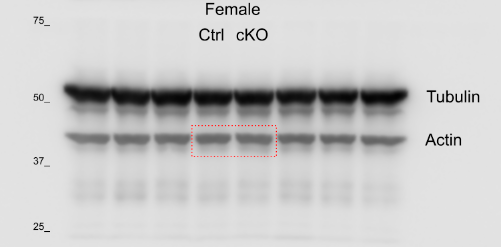
**

**Figure 5**

**A**

**
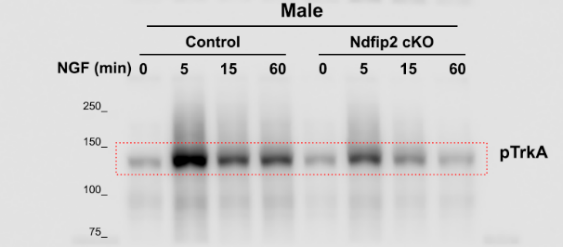

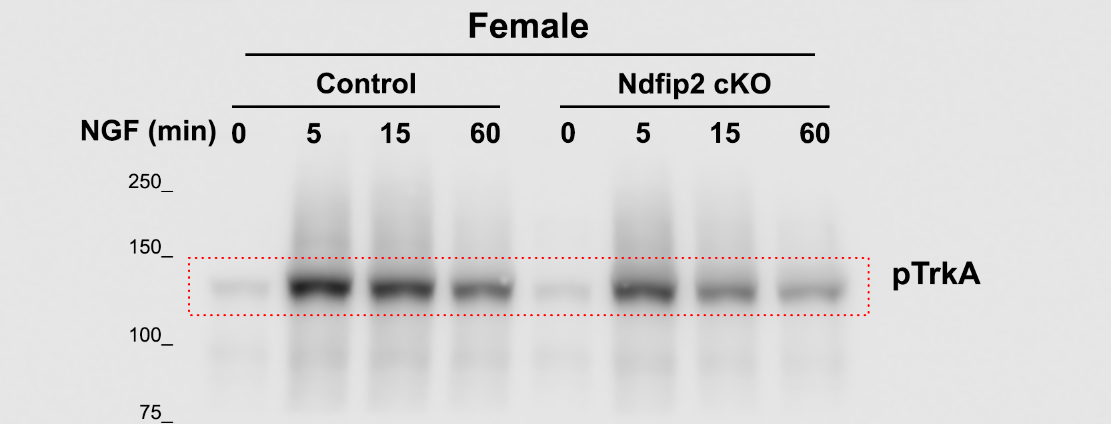
**

**
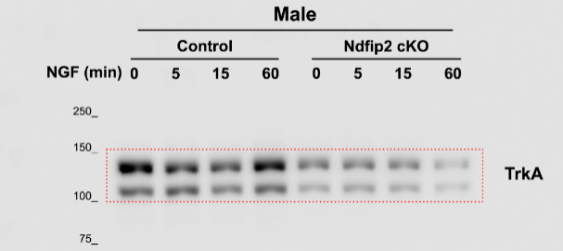

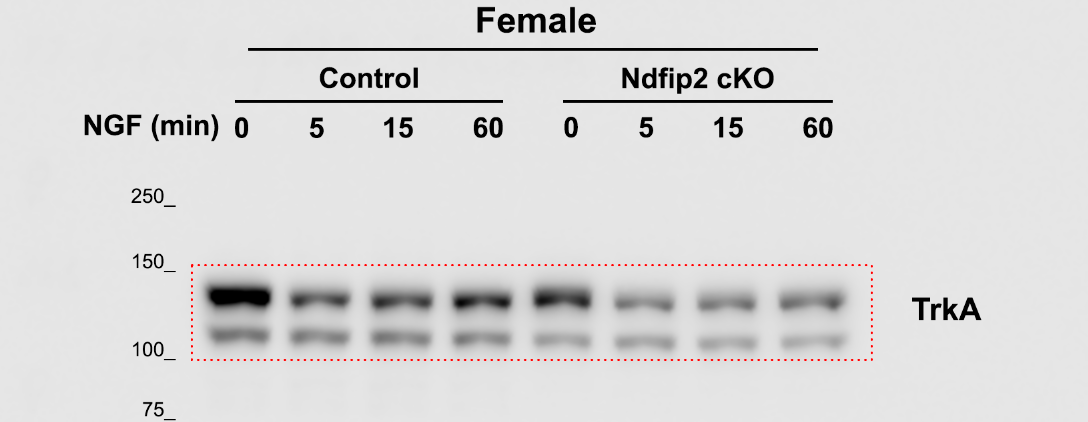
**

**
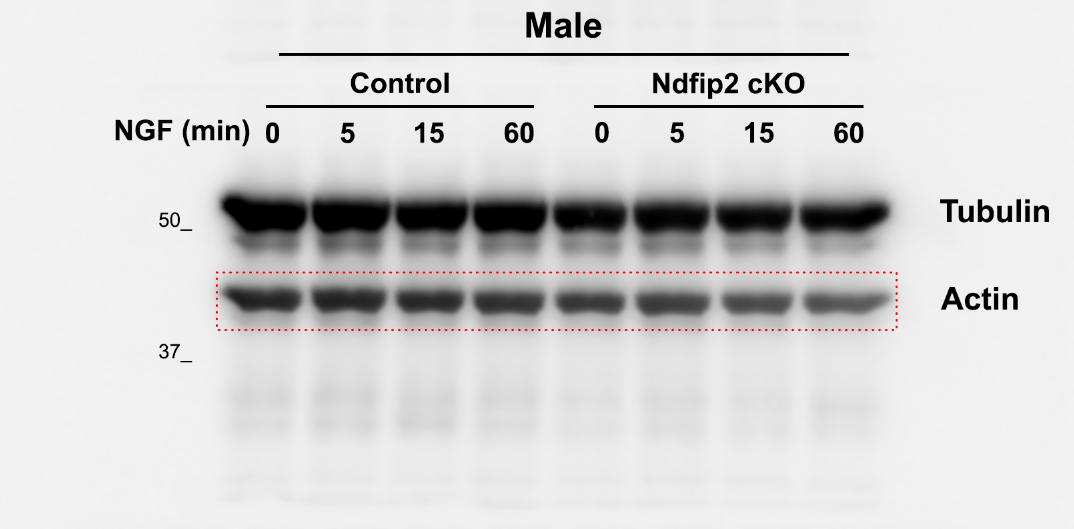

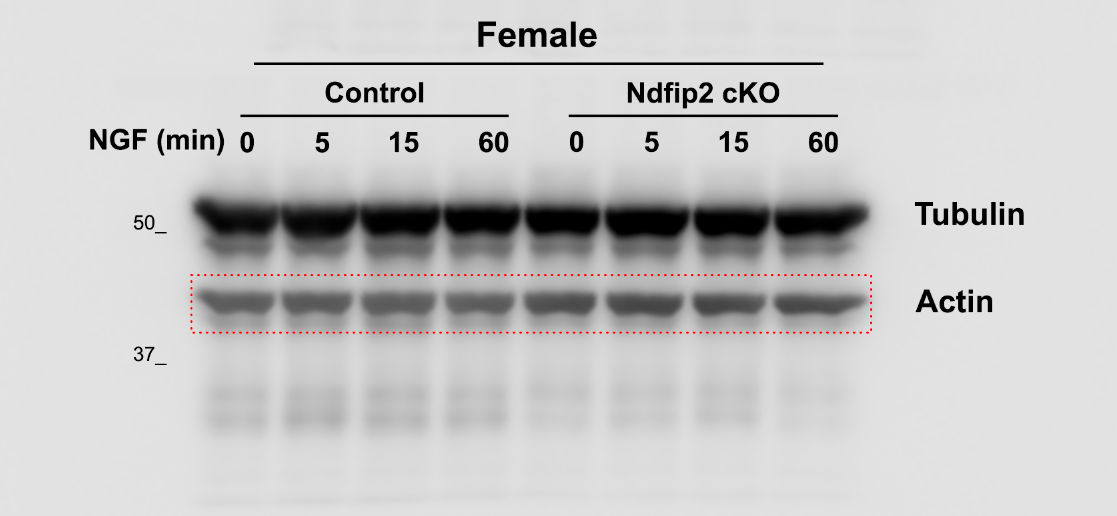
**

**
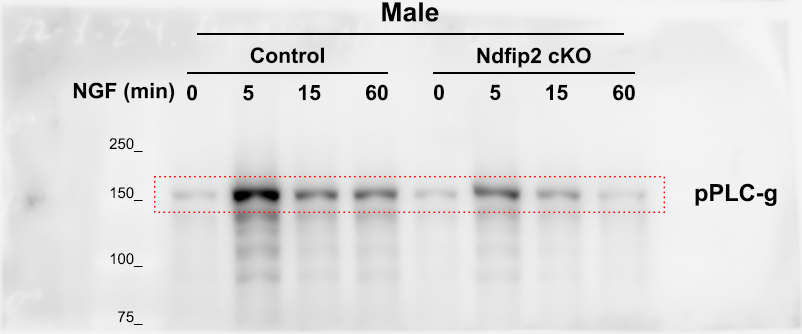

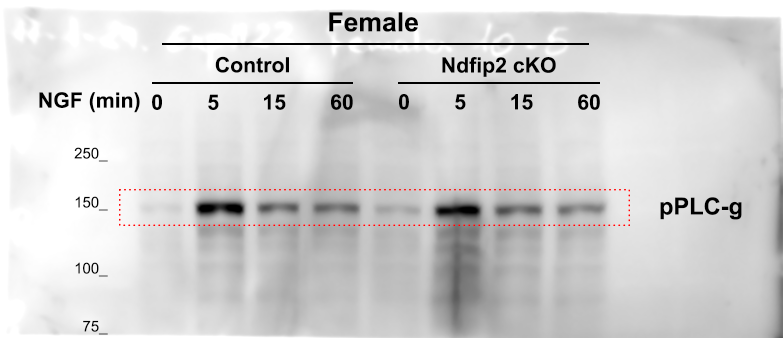
**

**
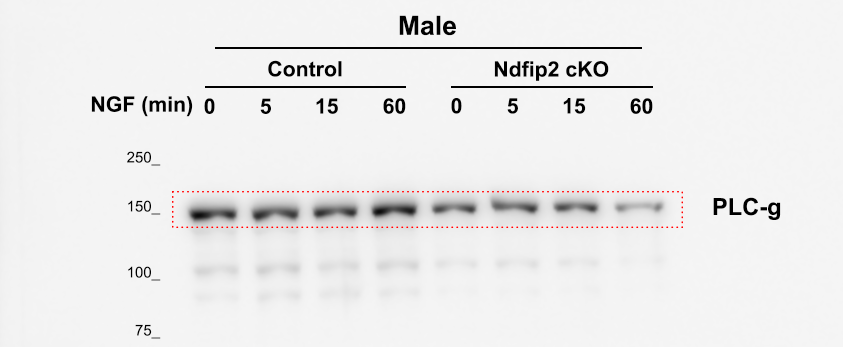

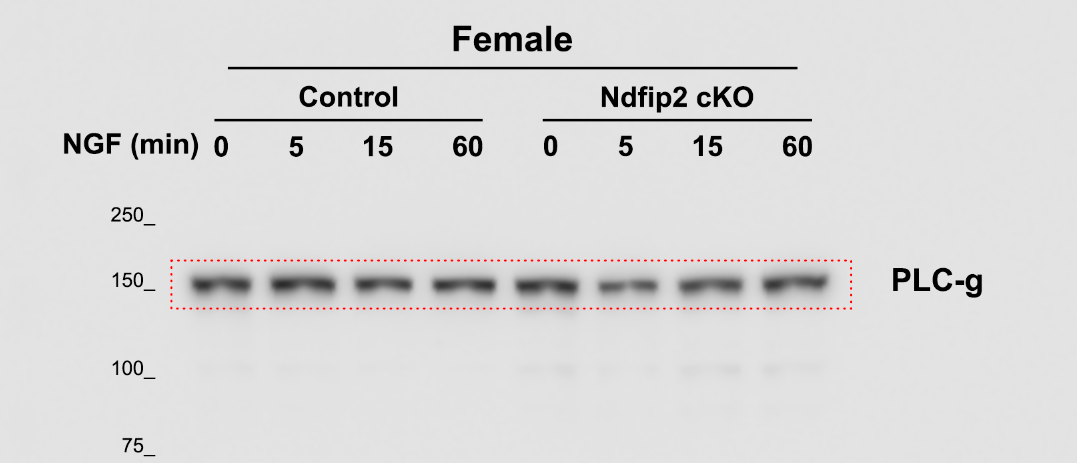
**

**
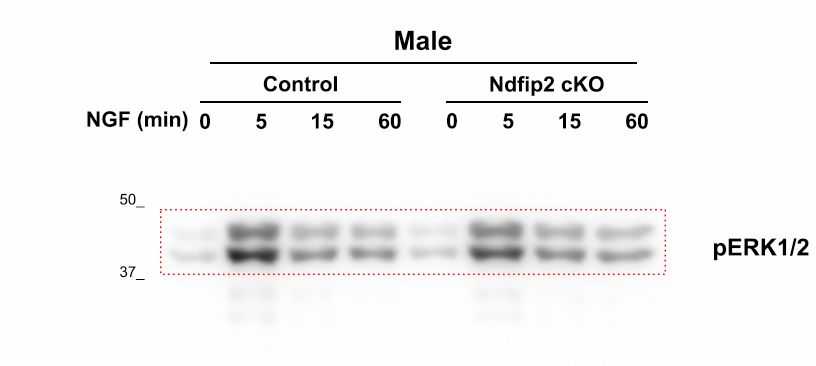

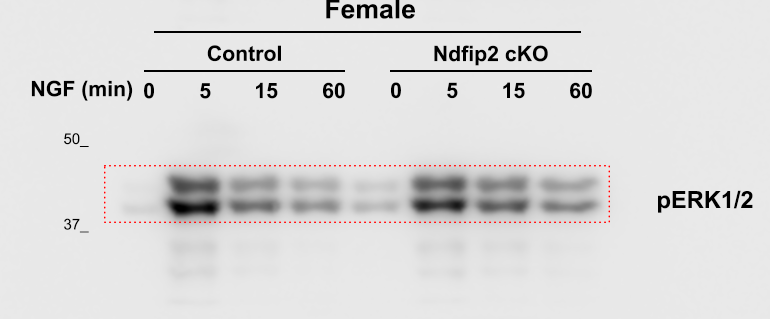
**

**
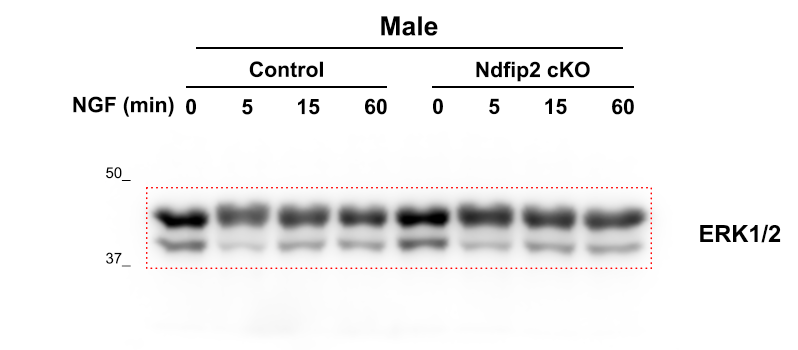

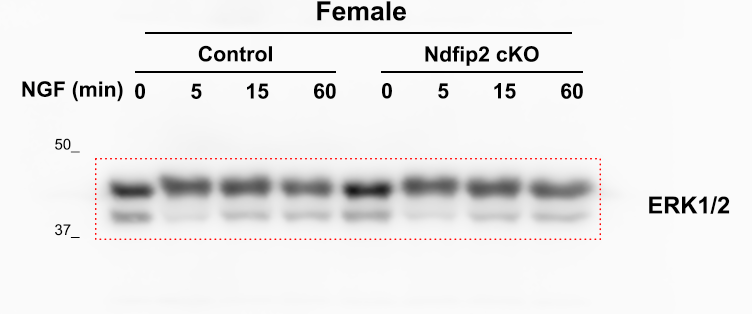
**

**
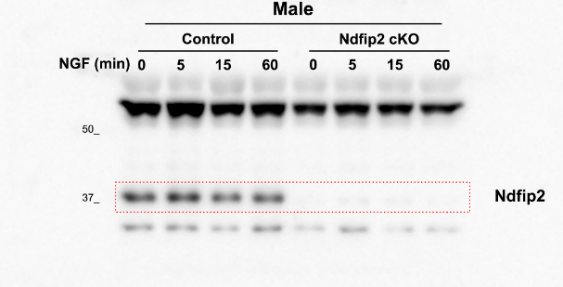
** **
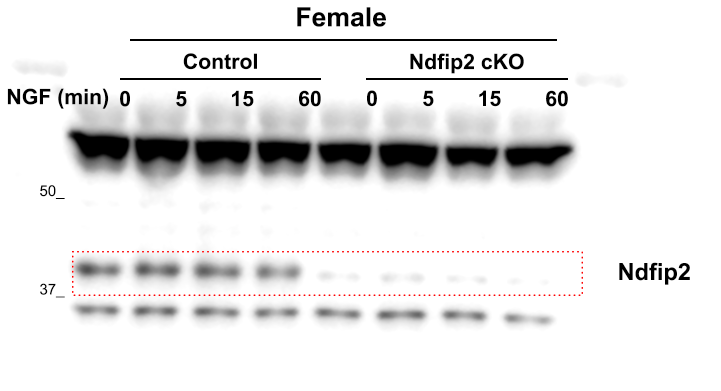
**

**I**

**
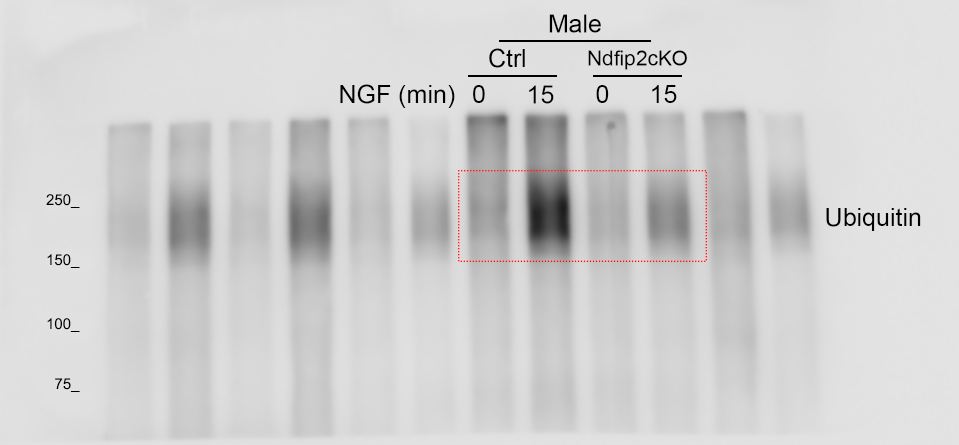

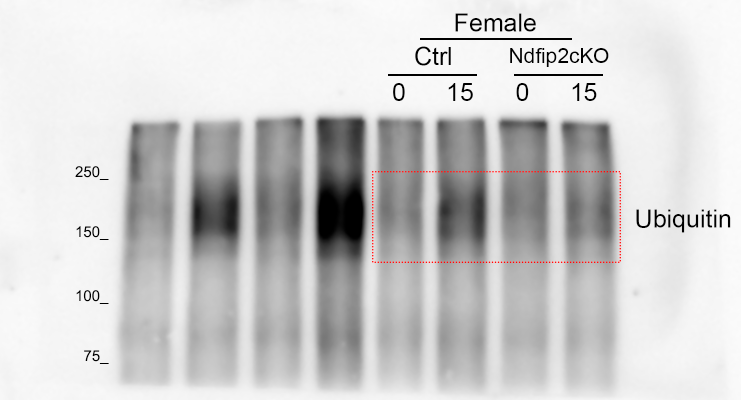
**

**
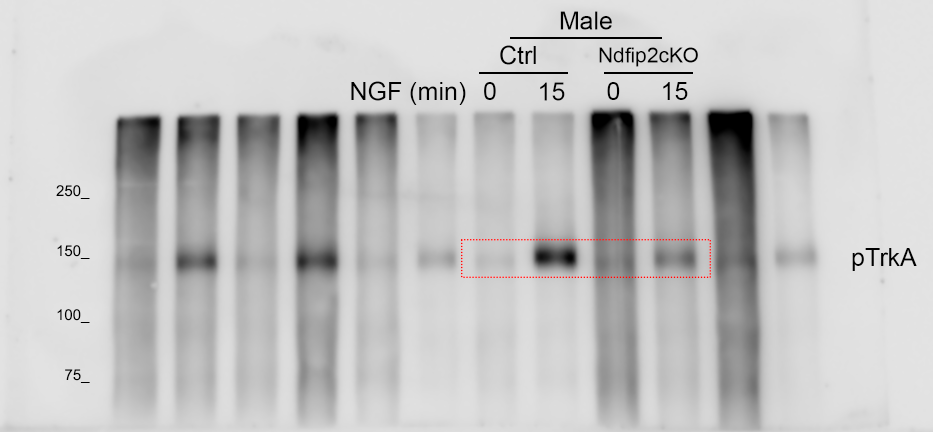

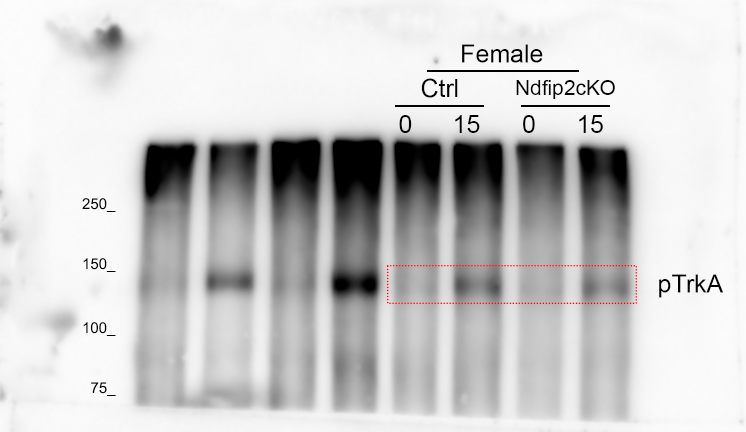
**

**
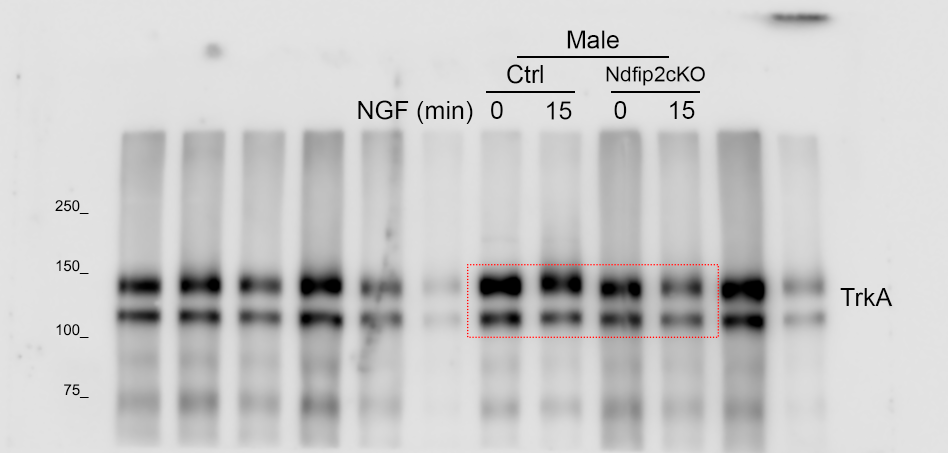

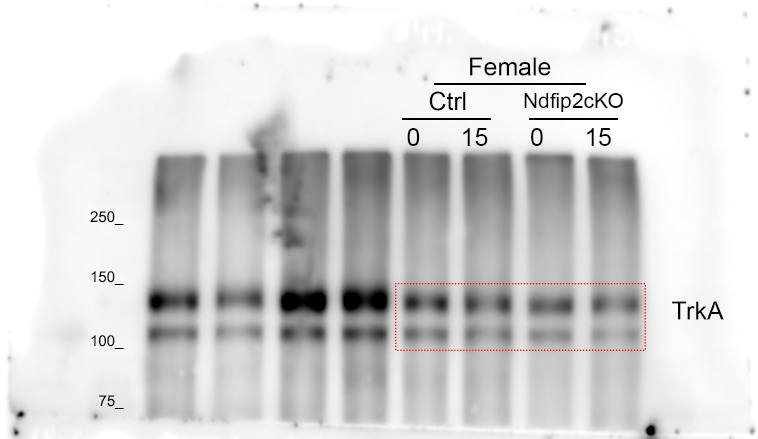
**

**K**

**
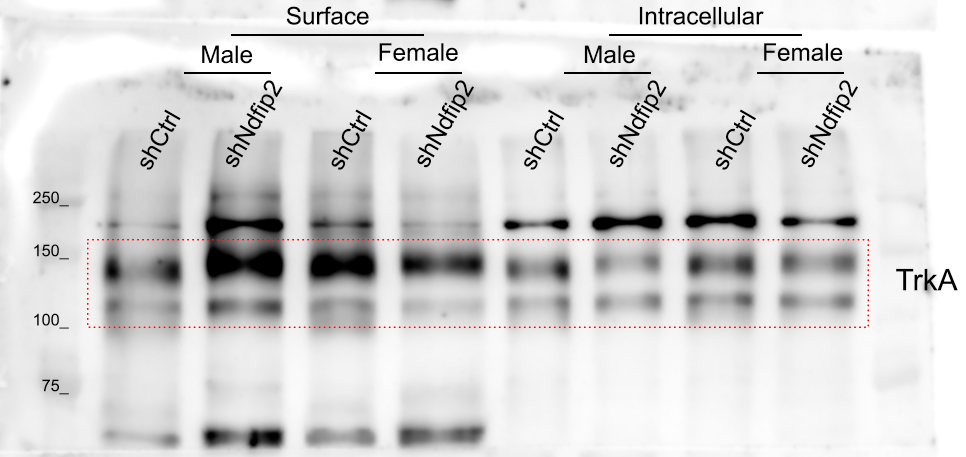
**

**
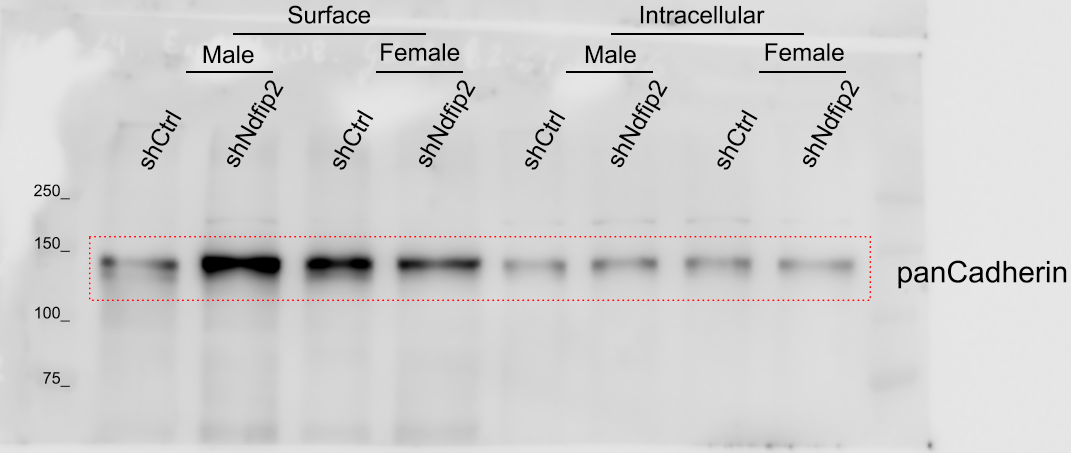
**

**
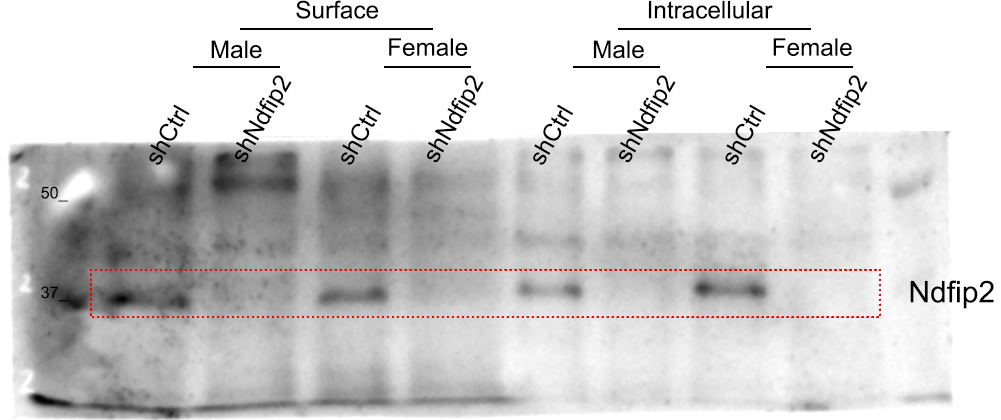
**

**M**


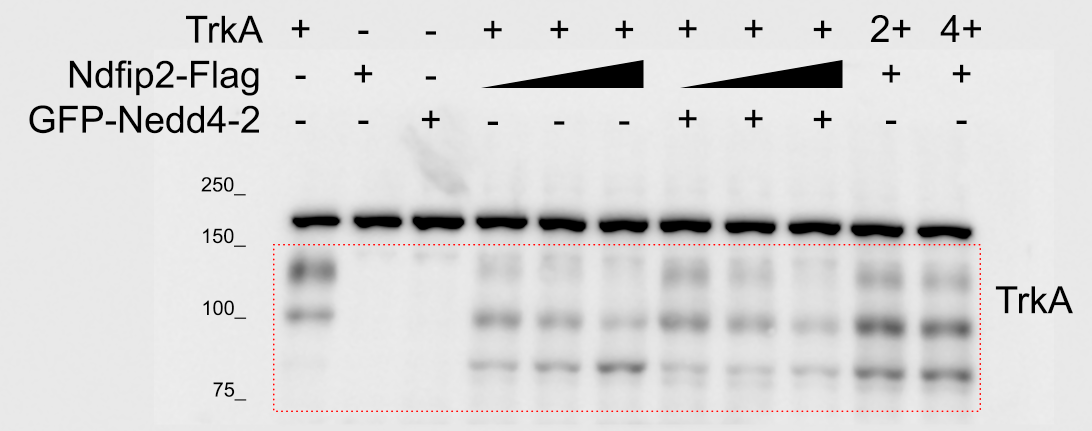


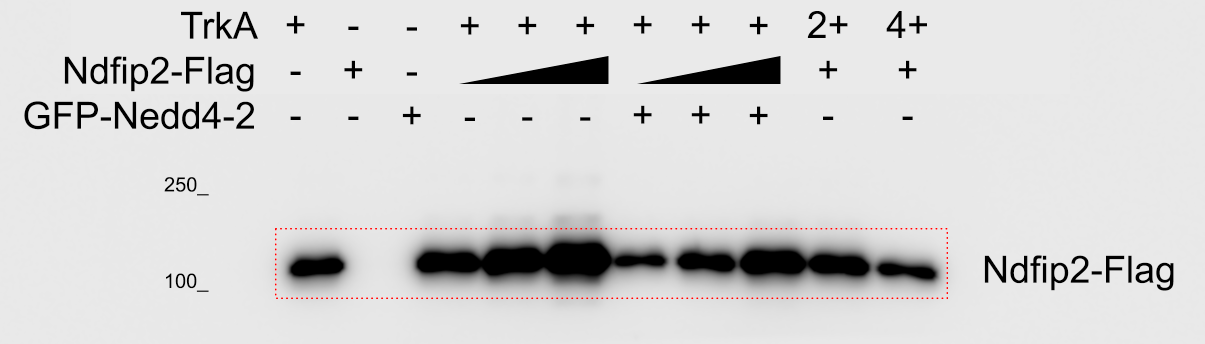


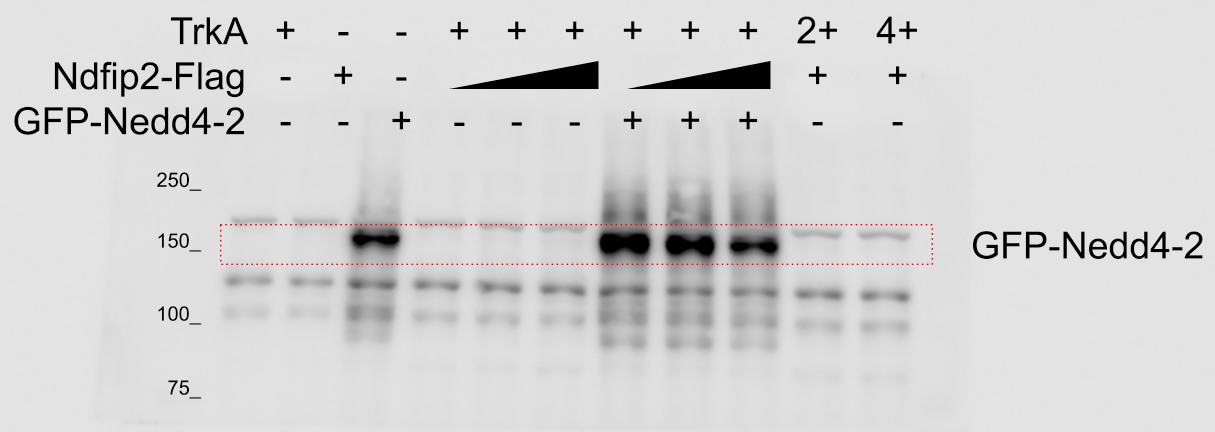


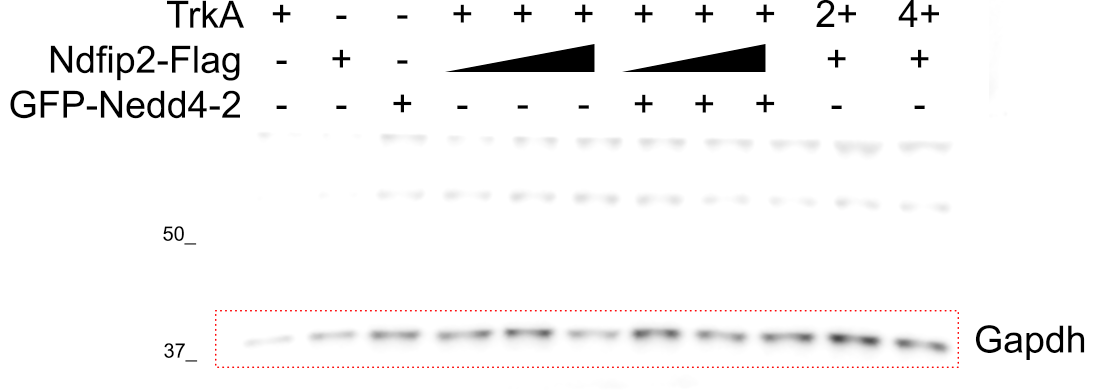

Supplement: Supplementary file 1 — Supplementary material [file 41419_2026_8670_MOESM1_ESM.docx]
